# Pheromone-Binding Protein 1 Performs a Dual Function for Intra- and Intersexual Signaling in a Moth

**DOI:** 10.3390/ijms252313125

**Published:** 2024-12-06

**Authors:** Yidi Zhan, Jiahui Zhang, Mengxian Xu, Frederic Francis, Yong Liu

**Affiliations:** 1 State Key Laboratory of Wheat Improvement, Shandong Agricultural University, No. 61, Daizong Road, Taian 271018, China; zhanyidi@sdau.edu.cn; 2 College of Plant Protection, Shandong Agricultural University, No. 61, Daizong Road, Taian 271018, China; zjh10120021@163.com (J.Z.); 13953865043@163.com (M.X.); frederic.francis@uliege.be (F.F.); 3 Functional and Evolutionary Entomology, Gembloux Agro-Bio Tech, Liege University, Passage des Deportes 2, 5030 Gembloux, Belgium

**Keywords:** intraspecies communication, pheromone recognition mechanism, mating behavioral manipulation

## Abstract

Moths use pheromones to ensure intraspecific communication. Nevertheless, few studies are focused on both intra- and intersexual communication based on pheromone recognition. Pheromone-binding proteins (PBPs) are generally believed pivotal for male moths in recognizing female pheromones. Our research revealed that PBP1 of *Agriphila aeneociliella* (AaenPBP1) serves a dual function in both intra- and intersexual pheromone recognition. Here, a total of 20 odorant-binding protein (OBP) family genes from *A. aeneociliella* were identified and subjected to transcriptional analysis. Among these, *AaenPBP1* was primarily highly expressed in the antennae. Competitive fluorescence binding assays and molecular docking analyses demonstrated that AaenPBP1 exhibits a strong binding affinity for the female sex pheromone (Z)-9-Hexadecenyl acetate and the male pheromone 1-Nonanal. Notably, hydrogen bonds were observed between Ser56 and the ligands. The analysis of pheromone components and PBPs in lepidopteran lineage suggested that their strong and precise interactions, shaped by coevolution, may play a crucial role in facilitating reproductive isolation in moths. Our findings provide valuable insight into the functional significance of PBPs in invertebrates and support the development of behavioral regulation tools as part of an integrated pest management strategy targeting crambid pests.

## 1. Introduction

Insects possess a highly sensitive olfactory system capable of accurately detecting and processing diverse odor signals, enabling them to perform critical behaviors such as host searching, locating spawning sites, mate finding, individual communication, and natural enemy avoidance [1,2,3]. The sex pheromone communication system of moths is one of the most typical examples of insect communication in which males often use smell to identify sex pheromones released by females to locate mates [4]. The complexity and sensitivity of moth sexual pheromone communication systems allow even subtle variations in pheromone signals to effectively maintain reproductive isolation in natural populations [5,6,7,8].

Intra- and intersexual pheromones are chemical signals released by male and female moths to facilitate intrasexual competition and mate selection, thereby optimizing mating success and population reproduction. While moths are typically considered to be attracted to mates through the detection of sex pheromones released by intersexual conspecifics, these pheromones can also regulate intrasexual behaviors. For instance, the male pheromone of *Conogethes punctiferalis* (Guenée) serves as a signal for conspecific male recognition and may function as an aphrodisiac [9]. Conversely, the male pheromone of the armyworm *Pseudaletia unipuncta* (Haw.) deters multiple males from competing for a single female [10]. Investigating the mechanisms underlying intra- and intersexual communication provides valuable insight into the sophisticated reproductive strategies of moths, including intraspecific reproduction and interspecific reproductive isolation.

The insect olfactory system involves multiple olfaction-related proteins that mediate the process of odor recognition [11]. In lepidopteran insects, classical odorant-binding proteins (OBPs) are typically classified into five subfamilies based on amino acid sequence homology: PBPs, GOBP1s (type 1 general odorant-binding proteins), GOBP2s (type 2 general odorant-binding proteins), ABP1s (type 1 antennal binding proteins), and ABP2s (type 2 antennal binding proteins) [12]. PBPs exhibit the hallmark characteristics of OBPs—they are acidic, water-soluble small proteins comprising 120–160 amino acids. Beyond their role as passive carriers that solubilize lipophilic pheromones in the hydrophilic antennal lymph, PBPs are hypothesized to contribute to the remarkable specificity of the insect olfactory system [13,14]. For instance, SfruPBP1 was reported to play a key role in sex pheromone discrimination, driving sex-specific behavioral responses to particular pheromone components in *Spodoptera frugiperda* [15]. Similarly, the OnubPBP3 complex exhibits greater stability with E-pheromone than with Z-pheromone in *Ostrinia nubilalis* [16]. In *Bombyx mori* (Lepidoptera: Bombycidae), BmPBP1 is essential for pheromone detection, as antennae exhibit significantly reduced electrophysiological sensitivity when BmPBP1 expression is knockdown [17,18].

The Eastern Grass Veneer moth, *Agriphila aeneociliella* (Eversmann) (Lepidoptera: Crambidae), is a devastating pest in wheat production, with its larvae feeding on the base of the wheat stalks and causing substantial yield losses during population outbreaks [19]. Previous studies have shown that the sex pheromones of *A. aeneociliella* regulate courtship and intrasexual competition [18]. Using electrophysiological, chemical, and olfactory behavioral assays, the female pheromones were identified as (Z,Z,Z)-9,12,15-Octadecatrienal and (Z)-9-Hexadecenyl acetate, while the male pheromone was determined to be 1-Nonanal [20]. Male moths select mates by detecting both female and male pheromones, but the underlying olfactory recognition mechanisms remain unclear. In this study, we screened and identified OBPs from the *A. aeneociliella* transcriptome and identified a key PBP through tissue-specific expression analysis. The binding properties of PBP1 to various pheromone components were characterized using molecular docking and in vitro binding assays. Additionally, the distribution and phylogeny relationships of PBP1 in lepidopteran insects were analyzed. This study provides insights into the mechanisms of sexual attraction and intrasexual competition in moths, offering potential strategies for pest management based on behavioral regulation.

## 2. Results

### 2.1. Identification and Characterization of OBP Genes

**Figure 1:**
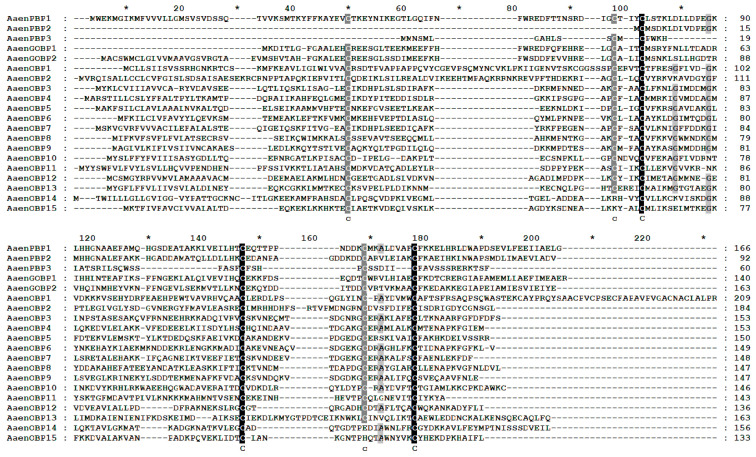
Multiple sequence alignment of *Agriphila aeneociliella* odorant-binding proteins (OBPs). Conserved amino acid residues are highlighted in black (highly conserved) and grayscale (moderately conserved). The asterisks indicate the count of amino acids.

**Figure 2:**
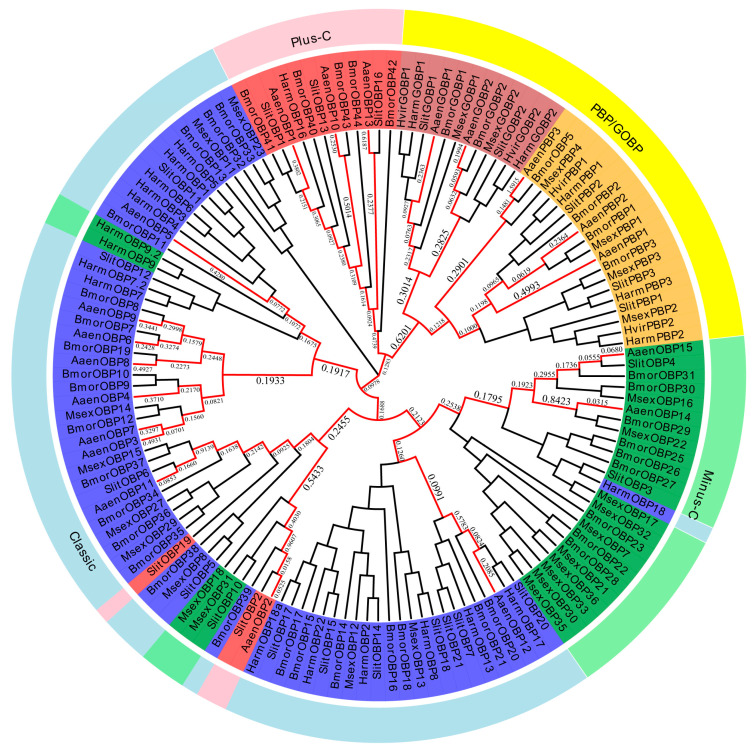
Phylogenetic analysis of odorant-binding proteins (OBPs) from *Agriphila aeneociliella* and other lepidopteran species. OBPs are categorized into subfamilies: typical OBPs (blue), Minus-C OBPs (green), Plus-C OBPs (red), and PBP/GOBP (yellow). Species abbreviations: Bmor (*Bombyx mori*), Slit (*Spodoptera littoralis*), Hvir (*Heliothis virescens*), Harm (*Helicoverpa armigera*), and Msex (*Manduca sexta*).

**Figure 3:**
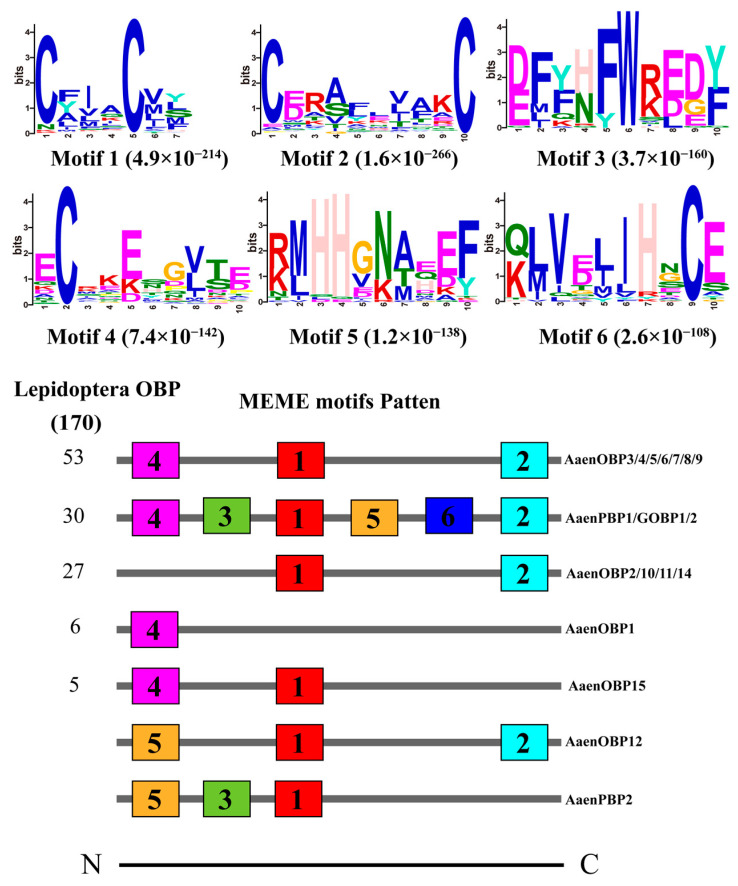
MEME motif pattern analysis of *Agriphila aeneociliella* odorant-binding proteins (OBPs). The upper section illustrated the six motifs identified in lepidopteran OBPs, with each motif represented by a numbered box. The lower section displays the most commonly occurring motif patterns, with the numbers in the boxes corresponding to the motifs shown in the upper section.

Based on functional annotation and tBLASTx results, a total of 20 putative OBP genes (*AaenPBP1-3*, *AaenGOBP1-2*, and *AaenOBP1-15*) were identified in *A. aeneociliella* (Table 1). Of these, 16 OBPs were obtained from larval transcriptomes and six from adult transcriptomes, with GOBP2 and OBP7 present in both stages (Figure S1). Based on the conserved cysteine residues identified through amino acid sequence alignment, the 20 OBPs were classified into three OBPs types: OBP14 and OBP15 were Minus-C OBPs; OBP1/2/10/13 were Plus-C OBPs; OBP3-9, OBP11, and OBP12 were Classic OBPs (Figure 1). A phylogenetic tree constructed using maximum likelihood analysis grouped the AaenOBPs with OBPs from other lepidopteran species. Most OBPs clustered into distinct subfamily groups, including PBP/GOBP, Classic, Plus-C, and Minus-C. PBP and GOBP sequences were highly conserved and formed clades based on their specialized functions (Figure 2). Using the MEME program, 53 of 170 OBPs analyzed displayed the common 4-1-2 motif pattern, while 30 exhibited the 4-3-1-6-5-2 motif pattern. Among them, motif 1 and motif 2 were highly conserved across lepidopteran OBPs (Figure 3).

### 2.2. Expression Profiles of A. aeneociliella OBPs

**Figure 4:**
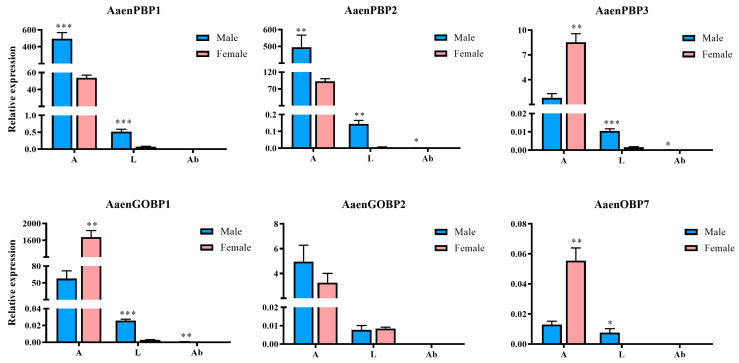
Transcript levels of odorant-binding protein (OBP) genes in various tissues of *Agriphila aeneociliella*. A: antennae; L: legs; Ab: abdomens. Data are presented as mean ± SE. Asterisks indicate statistically significant differences (*, *p* < 0.05; **, *p* < 0.01; ***, *p* < 0.001).

qRT-PCR analysis was performed to examine the expression patterns of six OBPs identified from the adult antennae and body transcriptome (Figure 4). Among these, *PBP1*, *PBP2*, and *GOBP1* were highly enriched in the antennae. Both *PBP1* and *PBP2* exhibited significant male-biased expression, while the expression levels of *PBP3*, *GOBP1*, and *OBP7* were significantly higher in female antennae than in male antennae. In the legs, *PBP1* and *PBP2* were expressed at higher levels compared to other genes and also displayed male-biased expression (Figure 4).

### 2.3. Binding Assays of Female and Male Pheromones to AaenPBP1

**Figure 5:**
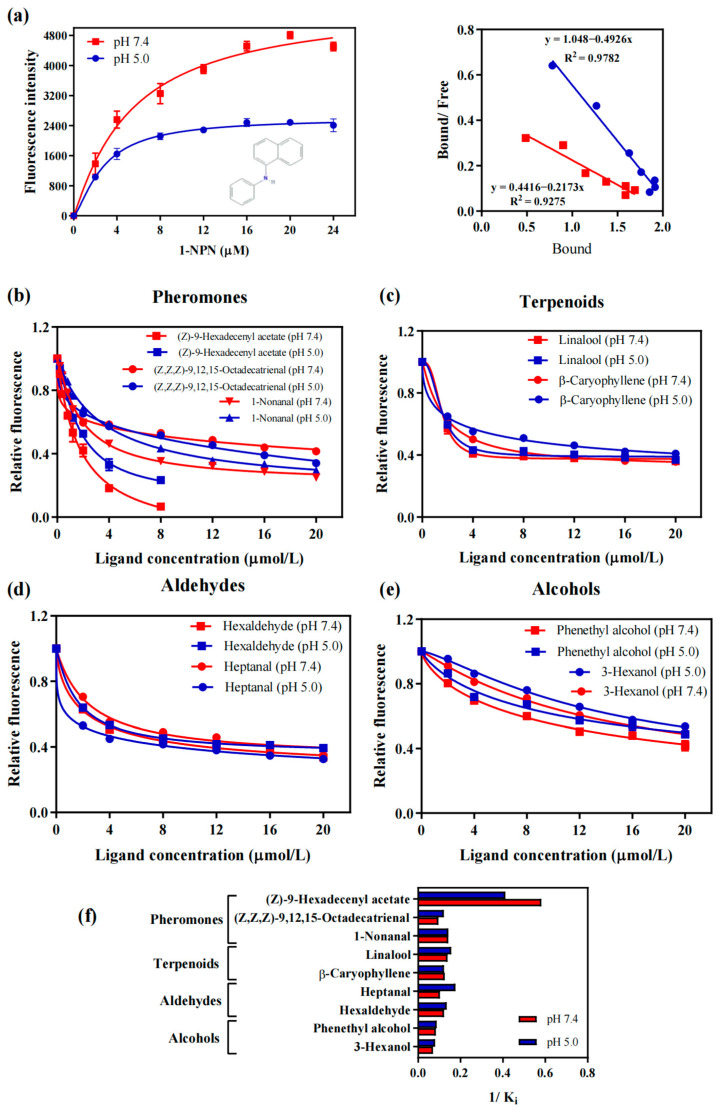
Competitive binding assays of AaenPBP1 to *Agriphila aeneociliella* male and female pheromones. (**a**) Binding curves and Scatchard plots of the probe 1-NPN to AaenPBP1 at pH 7.4 and 5.0. (**b**) Competitive binding properties of AaenPBP1 with female and male pheromones at pH 7.4 and 5.0. (**c**–**e**) Competitive binding curves of AaenPBP1 with six host-plant volatiles at pH 7.4 and 5.0: terpenoids (**c**), aldehyde (**d**), alcohols (**e**). (**f**) Comparison of the binding ability (1/Ki) of AaenPBP1 with three pheromones and six host-plant volatiles at pH 7.4 and 5.0.

High-yield recombinant AaenPBP1 protein was successfully obtained. SDS-PAGE analysis of the induction and purification process confirmed the presence of the recombinant AaenPBP1 protein, with a molecular weight of approximately 18.9 kDa (Figure S2). Competitive fluorescence binding assays were performed to assess the binding properties of AaenPBP1 with female and male pheromones, as well as host-plant volatiles. Scatchard analysis was conducted to determine the dissociation constant (*K*_d_), which was found to be 4.60 μM at pH 7.4 and 2.03 μM at pH 5.0 for 1-NPN (Figure 5a). The binding analysis revealed that both female and male pheromones interact with AaenPBP1 at both pH levels (Figure 5b). The protein preferentially bound to (Z)-9-Hexadecenyl acetate, with the lowest inhibition constant (*K*_i_) values of 1.72 μM at pH 7.4 and 2.43 μM at pH 5.0. PBP1 also exhibited relatively higher binding affinity for male pheromone 1-Nonanal, with *K*_i_ values of 7.04 μM at pH 7.4 and 7.00 μM at pH 5.0. Notably, AaenPBP1 displayed stronger affinities for (Z)-9-Hexadecenyl acetate and 1-Nonanal at lower concentrations but weaker binding to (Z,Z,Z)-9,12,15-Octadecatrienal at both pH levels (Figure 5b,f). Furthermore, PBP1 demonstrated general binding affinity for terpenoids and aldehydes from host-plant volatiles but weaker binding to alcohol-based volatiles (Figure 5c,d).

### 2.4. Molecular Docking of AaenPBP1 to Agriphila aeneociliella Sex Pheromones

**Figure 6:**
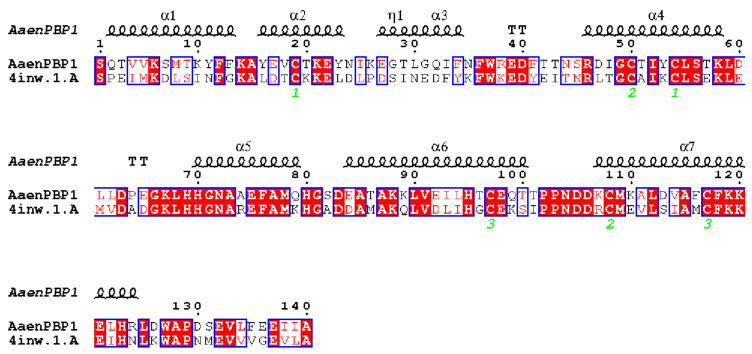
Sequence alignment of AaenPBP1 and AtraPBP1 pheromone-binding proteins. Conserved residues are highlighted, with the three disulfide bridges denoted by green numbers. The alignment highlights structural similarities between AaenPBP1 and the AtraPBP1 template (PDB ID: 4INW).

**Figure 7:**
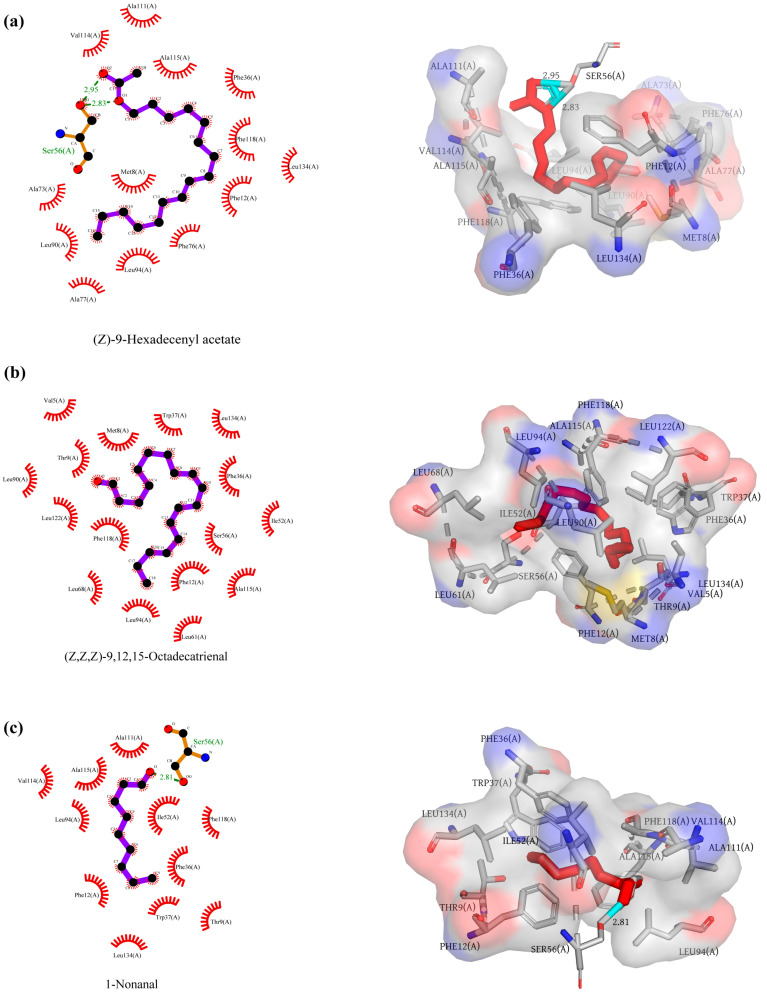
Molecular interactions of AaenPBP1 with two female components and one male pheromone component. The 2D and 3D interaction diagrams illustrate the binding of AaenPBP1 with (Z)-9-Hexadecenyl acetate (**a**), (Z,Z,Z)-9,12,15-Octadecatrienal (**b**), and 1-Nonanal (**c**). Hydrogen bonds and hydrophobic interactions with specific amino acid residues are labeled. The distances of the hydrogen bonds are indicated in (**a**) and (**c**).

To elucidate the binding mechanism of AaenPBP1 with female and male pheromones, molecular docking was performed using SYBYL X. The 3D structure of AaenPBP1 was built using AtraPBP1 as the template (Figure 6), and the molecular docking results are listed in Table S4. The docking results revealed that AaenPBP1 exhibits strong binding affinity with (Z)-9-Hexadecenyl acetate and 1-Nonanal. (Z)-9-Hexadecenyl acetate formed a tight interaction within the AaenPBP1 binding pocket, stabilized by hydrogen bonds and hydrophobic interactions. Specifically, the O1 and O2 atoms of (Z)-9-Hexadecenyl acetate formed hydrogen bonds with Ser56 of AaenPBP1 (Figure 7a). Similarly, the oxygen atom of 1-Nonanal was stabilized by a hydrogen bond with Ser56 (Figure 7c). In contrast, the binding of (Z,Z,Z)-9,12,15-Octadecatrienal relied predominantly on hydrophobic interactions (Figure 7b).

### 2.5. Phylogenetic Analysis

**Figure 8:**
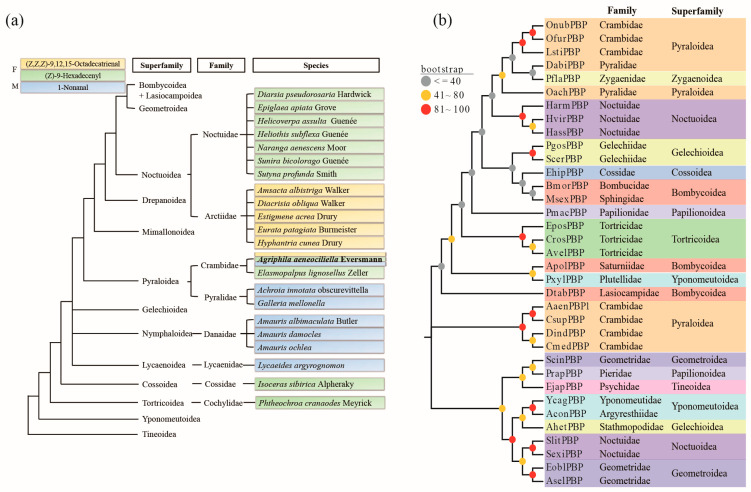
Distribution of pheromones and phylogenetic analysis of PBPs in moths and butterflies. (**a**) The presence and utilization of three pheromones—(Z,Z,Z)-9,12,15-Octadecatrienal, (Z)-9-Hexadecenyl acetate, and 1-Nonanal—across moths and butterflies. “F” represents female sex pheromones, and “M” represents male sex pheromones. The tree topology follows Mitter et al. [21]. (**b**) Phylogenetic tree depicting the relationships of PBPs from various moths and butterflies, including *Agriphila aeneociliella*. Detailed information about the PBPs and pheromones for each species is provided in Table S5.

In moths and butterflies, some species use one of three chemicals—(Z,Z,Z)-9,12,15-Octadecatrienal, (Z)-9-Hexadecenyl acetate, and 1-Nonanal—as female or male pheromones. Apart from *A. aeneociliella*, five moth species use (Z,Z,Z)-9,12,15-Octadecatrienal, and ten moth species use (Z)-9-Hexadecenyl acetate as female pheromone. Meanwhile, two moth species and four butterfly species employ 1-Nonanal as a male pheromone (Figure 8a). Among these, (Z,Z,Z)-9,12,15-Octadecatrienal has been identified as a female pheromone in five species of the family Arctiidae, while (Z)-9-Hexadecenyl acetate serve as a female pheromone in seven species of the family Noctuidae. Remarkably, *Elasmopalpus lignosellus* Zeller, a member of the same family as *A. aeneociliella*, also utilizes (Z)-9-Hexadecenyl acetate as one of its female pheromone components. The comparative phylogenetic analysis of PBPs in lepidopteran species (Figure 8b) places AaenPBP1 within a clade of PBPs from three Crambidae moths (*Chilo suppressalis*, *Diaphania indica*, and *Cnaphalocrocis medinalis*). All four moths are type I pheromone users whose sex pheromones include aldehydes and/or acetate (Table S5).

## 3. Discussion

An efficient olfactory system is crucial for insects to perform vital behaviors such as mating, foraging, avoiding enemies, and reproducing [22]. OBPs are believed to mediate the recognition and transport of external odors, playing pivotal roles in various behavioral responses. The higher number of chemosensory genes identified in larvae compared to adults suggests that these genes may have stage-specific functions during growth and development. Quantitative PCR revealed that the expression levels of PBP1 and PBP2 in male antennae were nearly 10 times higher than in female antennae. Similarly, those two genes showed significantly higher expression in male legs compared to female legs. These findings suggest that PBP1 and PBP2 play crucial roles in male recognition of female pheromones. OBPs represent a diverse protein family playing multifunctional roles beyond chemical detection. Their structural simplicity and stability allow them to adapt to various functions [23]. In non-sensory organs, OBPs and PBPs are often implicated in storing pheromones within specific glands and gradually releasing them into the environment. Structurally similar or even identical OBPs can participate both in detecting signaling chemicals in sensory organs and transmitting them from secretory glands [23]. For example, the relative expression of *AaenPBP2* was significantly higher in male antennae and abdomens than in their female counterparts, suggesting that AaenPBP2 participates not only in the perception of sex pheromone but also in the secretion of male pheromone. Additionally, OBPs and PBPs in pheromone-secreting glands might regulate the relative concentration of pheromone components based on specific temporal needs [24]. This mechanism may offer an efficient alternative for insects, as regulating the expression of a single protein requires activating only one gene, whereas pheromone synthesis typically involves multiple enzymes.

The binding selectivity and affinity of PBPs are important for understanding the specificity and sensitivity of pheromone perception in insects. Consequently, many studies have focused on the in vitro binding analysis of moth PBPs. To preliminarily investigate the pheromone perception mechanism of *A. aeneociliella* toward three identified pheromones, AaenPBP1, which is highly expressed in both male and female antennae, was selected for prokaryotic expression. At a neutral pH, AaenPBP1 showed stronger binding to (Z)-9-Hexadecenyl acetate and 1-Nonanal than at an acidic pH, indicating a pH-dependent conformational change mechanism [25,26,27]. AtraPBP1 was selected as the structural template for modeling AaenPBP1, with a sequence identity of 48.57%. The AaenPBP1 structure comprises seven α-helices, consistent with BmorPBP [28]. Three disulfide bonds formed by six conserved cysteine residues stabilize the helices, connecting α2 and α4 (Cys19-Cys54), α4 and α7 (Cys50-Cys108), α6 and α7 (Cys97-Cys117) (Figure 6). The molecular docking results align with the fluorescence competitive binding assays, further confirming strong interactions between AaenPBP1 and (Z)-9-Hexadecenyl acetate as well as 1-Nonanal. The presence of hydrogen bonds involving Ser56 highlights the crucial role of this conserved residue in pheromone recognition [28].

*A. aeneociliella* exemplifies a typical type I pheromone user, utilizing (Z,Z,Z)-9,12,15-Octadecatrienal and (Z)-9-Hexadecenyl acetate individually or in combination. Interestingly, most Noctuoidea insects rely on these two components as pheromones. While most type I pheromones are biosynthesized from saturated fatty-acyl CoA precursors, type II pheromones typically derive from dietary linoleic or linolenic acid [27]. Despite being classified as a type I pheromone due to its terminal functional group, (Z,Z,Z)-9,12,15-Octadecatrienal originates from α-linolenic acid, resembling the synthesis pathway of type II pheromones [29,30]. Thus, *A. aeneociliella* appears to possess both type I and type II pheromone synthesis pathways, resembling evolutionary patterns observed in more advanced Noctuoidea. However, only one Pyraloidea species, *Elasmopalpus lignosellus* Zeller, shares the pheromone component (Z)-9-Hexadecenyl acetate with *A. aeneociliella*. Selection pressures likely favor emitters reshuffling enzymatic pathways to generate novel pheromone blends and receivers evolving broader sensory capabilities to detect new components [31]. Strong selection for species-specific recognition [32] or reinforcement to avoid hybridization with sympatric species [33] results in reproductive character displacement, such as the unique sex pheromones of *A. aeneociliella* [31].

## 4. Materials and Methods

### 4.1. Insect Rearing

A laboratory colony of *A. aeneociliella* was established, with larvae reared on wheat seedlings under controlled conditions of 24.0 ± 0.5 °C, 75 ± 5% relative humidity, and a 12 h light/12 h dark photoperiod. Last-instar larvae were individually placed in 30 mL plastic cups containing sterilized fine vermiculite (moisture content 20%) for pupation until adult emergence.

### 4.2. RNA Isolation, cDNA Synthesis, and Illumina Sequencing

Larvae and adults of *A. aeneociliella* (a mixture of males and females) were rapidly separated and stored in liquid nitrogen for subsequent RNA extraction. Total RNA was extracted using Trizol reagent (Invitrogen, Waltham, MA, USA) following manufacturer’s instruction. RNA quality and concentration were assessed with a NanoPhotometer^®^ spectrophotometer (Implen, Westlake Village, CA, USA) and the Qubit^®^ RNA Assay Kit with a Qubit^®^ 2.0 Fluorometer (Life Technologies, Carlsbad, CA, USA), respectively. cDNA library construction was carried out using the TruseqTM RNA sample prep Kit (Illumina, San Diego, CA, USA) and sequenced on an Illumina HiSeqTM 2000 platform (Illumina, San Diego, CA, USA). After removing low-quality reads and adapter sequences, de novo transcriptome assembly was performed using the Trinity program. The resulting unigenes were annotated by blasting against multiple databases, including non-redundant protein (Nr), nucleotide (Nt), Swiss-Prot, Clusters of Orthologous Groups (COG), Kyoto Encyclopedia of Genes and Genomes (KEGG), and Gene Ontology (GO).

### 4.3. Gene Identification and Bioinformatic Analysis

Putative OBP-related unigenes from the larval and adult transcriptomes of *A. aeneociliella* were identified using the tBLASTn program and manually confirmed through a BLASTx search on NCBI. Open reading frames (ORFs) were predicted with ORF Finder (https://www.ncbi.nlm.nih.gov/orffinder/; accessed on 7 July 2021), and N-terminal signal peptides were identified using SignalP-5.0 (https://services.healthtech.dtu.dk/services/SignalP-5.0/; accessed on 7 July 2021) [34]. Multiple sequence alignments were performed using Clustal X 2.0 [35], and phylogenetic trees were constructed with the maximum likelihood method in MEGA 7.0, evaluated using 1000 bootstrap replicates [36]. The final phylogenetic trees were visualized using EvolView (https://www.evolgenius.info/evolview/; accessed on 12 July 2021) [37]. All amino acid sequences are listed in Table S1.

For motif pattern discovery, 170 OBPs from lepidopteran insects were analyzed using the MEME online server (version 5.3.3, http://meme-suite.org; accessed on 13 July 2021) [38]. The motif discovery parameters were set to a minimum width of 6, a maximum width of 10, and a maximum of 8 motifs. All amino acid sequences are provided in Table S2.

### 4.4. Tissue Expression Profile Analysis

The expression levels of OBP genes in different tissues were assessed using quantitative real-time PCR (qRT-PCR). Antennae (A), legs (L), and abdomens (Ab) were collected separately from virgin male and female moths. RNA extraction and detection followed the previously described methods. cDNA templates were synthesized using PrimeScript II RTase with Oligo (dT) and Random 6 primer (PrimeScript™ II 1st Strand cDNA Synthesis Kit, Takara Bio, Beijing, China). *GAPDH* served as the reference gene for normalization. Specific qRT-PCR primers for adult-expressed genes were designed using Primer Premier 5.0 and are listed in Table S3. qRT-PCR reactions were performed on a CFX-96 Real-Time PCR Detection System (Bio-Rad, Hercules, CA, USA) using 2× T5 Fast qPCR Mix (TSINGKE, Qingdao, China). The reaction conditions were as follows: an initial denaturation at 95 °C for 1 min, followed by 40 cycles of 95 °C for 10 s, and a melting curve analysis at 60 °C to 65 °C with 0.55 °C increments for 15 s. Each sample included three technical and three biological replicates. Relative gene expression levels were calculated using the 2^−ΔΔCt^ method [39]. Comparative expression analyses between sexes were conducted using a *t*-test.

### 4.5. Preparation of Recombinant Pheromone-Binding Protein 1

Specific primers of *A. aeneociliella* pheromone-binding protein 1 (*AaenPBP1*) were designed and are listed in Table S3. PCR amplification was conducted using 2× Accurate Taq Master Mix (Accurate Biology Co., Ltd., Hunan, China) under the following conditions: an initial denaturation at 98 °C for 3 min, followed by 35 cycles of denaturation at 98 °C for 10 s, annealing at 45–72 °C for 30 s, extension at 72 °C for 1 min, and a final extension at 72 °C for 10 min. The PCR products were purified using a Gel Extraction Kit (Beijing CoWin Biotech Co., Ltd., Beijing, China) and subsequently ligated into the pMD18-T vector (Takara Bio, Beijing, China). The recombinant plasmids were transformed into *Escherichia coli* DH5α-competent cells and sequenced by TSINGKE (Qingdao, China) using the Sanger method.

The *AaenPBP1* gene, excluding the signal peptide, was amplified, and the purified product was cloned into the gene-15bs expression vector (pET-15b with a SUMO tag). The recombinant plasmid was transformed into *E. coli* BL21 (DE3)-competent cells, and positive clones were verified through sequencing. A confirmed single clone was inoculated into 2 L of LB medium at 37 °C until the OD_600_ reached 0.6. Protein expression was induced at 16 °C with shaking 160 rpm using 0.5 mM isopropyl-β-d-thiogalactopryranoside (IPTG). Bacterial cells were harvested by centrifugation (10,000 rpm, 10 min) and resuspended in 1 × phosphate-buffered saline (PBS). The cell suspension was disrupted via ultrasonication, and the resulting supernatant and precipitate were separately by centrifugation. SDS-PAGE analysis showed that the AaenPBP1 protein was present in the supernatant. The protein was purified using a Ni^2+^-NTA column (His-tagged purification) with gradient imidazole elution. Purity was assessed by SDS-PAGE, and the protein was subsequently dialyzed against PBS. Finally, the protein was dissolved in buffer solutions (50 mM Tris-HCL, pH 7.4 and 5.0) to a final concentration of 2 μM.

### 4.6. Fluorescence Binding Assay

Fluorescence binding assays were performed to assess the affinity of AaenPBP1 for three sex pheromones (two female and one male pheromones) and six host-plant volatile compounds (Table S4). Fluorescence spectra were recorded using an F-4600 FL spectrophotometer (Hitachi High-Tech Co., Ltd., Shanghai, China) with a 1 cm path-length quartz cuvette. The fluorescent probe N-phenyl-1-naphthylamine (1-NPN) was dissolved in methanol. Excitation was set at 337 nm, and emission spectra were recorded over a range of 350–600 nm. AaenPBP1 solutions (2 μM) at pH 7.4 and 5.0 were titrated with each ligand at a final concentration ranging from 2 to 24 μM. The half-maximal inhibitory concentration (IC_50_) values were determined through data linearization, and the dissociation constants (K_i_) of the competing ligands were calculated following the equation: *K*_i_ = [IC_50_]/(1 + [1-NPN]/*K*_1-NPN_), where [1-NPN] represents the free concentration of 1-NPN, and *K*_1-NPN_ is the dissociation constant of the protein/1-NPN complex [40]. Data analysis was conducted using GraphPad Prism 7.0 (GraphPad Software, Inc., San Diego, CA, USA).

### 4.7. Homology Modeling and Molecular Docking

The Protein Data Bank (PDB; http://www.rcsb.org; accessed on 21 August 2021)) was used to conduct a BLAST search of the AaenPBP1 amino acid sequence to identify suitable structural templates. Homology modeling was performed using the SWISS-MODEL server (https://swissmodel.expasy.org/interactive; accessed on 21 August 2021) [41]. The pheromone-binding protein 1 structure from *Amyelois transitella* (AtraPBP1, PDB ID: 4INW) was selected as the optional template. The quality of the generated 3D model was evaluated using PROCHECK, Verify 3D, and ERRAT methods. Molecular conformations of the ligands were obtained from Pubchem (https://pubchem.ncbi.nlm.nih.gov/; accessed on 23 August 2021) and ChemSpider (http://www.chemspider.com/; accessed on 23 August 2021). Molecular docking simulations were carried out using SYBYL-X 2.1.1 [42], with energy minimization performed via the Tripos force field and Gasteiger–Hückel charge methods. Binding affinity between AaenPBP1 and the ligands was assessed based on the total score. Protein–ligand interaction diagrams were generated in 2D using LigPlot^+^ v.2.2 [43], while 3D structural visualizations were created with PyMOL 2.2.0 (Pymol Molecular Graphics System, Schrodinger, LLC, New York, NY, USA).

## 5. Conclusions

Deeply uncovering the molecular mechanism of moth communication, this study reveals AaenPBP1 as a pivotal agent in both sexual attraction and intrasexual competition, challenging conventional views on pheromone-binding proteins (PBPs). By identifying its male-biased expression in *A. aeneociliella* antennae and its dual recognition of key female and male pheromone components, we demonstrated the multifunctional significance of PBP in lepidopteran insects.

## Figures and Tables

**Table 1:** Characterization and BLASTx matches of OBP genes identified in *Agriphila aeneociliella*.

Gene Name	ORF (aa)	Signal Peptide	Blastx Annotation	Acc. Number	Score	E-Value	Identity (%)
AaenPBP1	166	1–23	Pheromone-binding protein 1 [*Chilo suppressalis*]	ADK66921.1	250	2 × e^−82^	71%
AaenPBP2	3’miss	ND	Pheromone-binding protein 2 [*C. suppressalis*]	ACJ07123.1	150	2 × e^−44^	77%
AaenPBP3	60	ND	Pheromone-binding protein 3 [*C. suppressalis*]	ADL09140.1	93.6	2 × e^−22^	77%
AaenGOBP1	140	ND	General odorant-binding protein 1 [*C. suppressalis*]	ACJ07126.1	240	5 × e^−79^	80%
AaenGOBP2	163	1–21	General odorant-binding protein 2 [*C. suppressalis*]	ACJ07120.1	279	4 × e^−94^	81%
AaenOBP1	212	1–39	Odorant-binding protein 4 [*C. suppressalis*]	ANZ73034.1	229	2 × e^−73^	54%
AaenOBP2	184	1–22	Odorant-binding protein 18 [*Conogethes punctiferalis*]	QEE82717.1	368	1 × e^−128^	96%
AaenOBP3	153	1–19	Odorant-binding protein 25 [*C. suppressalis*]	ANC68513.1	201	9 × e^−63^	67%
AaenOBP4	153	1–23	Odorant-binding protein 13 [*Cnaphalocrocis medinalis*]	ALT31643.1	235	7 × e^−77^	74%
AaenOBP5	150	1–20	Odorant-binding protein [*C. suppressalis*]	AGM38610.1	167	3 × e^−50^	61%
AaenOBP6	149	1–19	Odorant-binding protein 22, partial [*C. suppressalis*]	ANC68510.1	191	7 × e^−59^	66%
AaenOBP7	148	1–20	Odorant-binding protein 29, partial [*C. suppressalis*]	ANC68517.1	213	2 × e^−68^	73%
AaenOBP8	147	1–18	Odorant-binding protein, partial [*C. punctiferalis*]	APG32537.1	149	2 × e^−43^	51%
AaenOBP9	147	1–20	General odorant-binding protein 28a [*Helicoverpa armigera*]	XP_021194660.1	166	1 × e^−49^	53%
AaenOBP10	146	1–17	Odorant-binding protein 4 [*C. suppressalis*]	AGK24580.1	264	1 × e^−88^	82%
AaenOBP11	3’miss	1–21	Odorant-binding protein 40 [*Dendrolimus punctatus*]	ARO70199.1	157	3 × e^−47^	68%
AaenOBP12	136	1–21	Odorant-binding protein [*C. suppressalis*]	AGM38607.1	236	3 × e^−78^	85%
AaenOBP13	163	1–16	Odorant-binding protein OBP47 [*Lobesia botrana*]	AXF48744.1	89.7	2 × e^−19^	37%
AaenOBP14	156	1–18	Odorant-binding protein 9 [*C. pinicolalis*]	QEE82708.1	247	1 × e^−81^	83%
AaenOBP15	133	1–16	Odorant-binding protein 2 [*C. suppressalis*]	AGK24578.1	254	3 × e^−85^	94%

ND: not detected.

## Data Availability

The original contributions presented in the study are included in the article; further inquiries can be directed to the corresponding author.

## Supplementary Materials

The following supporting information can be downloaded at: https://www.mdpi.com/article/10.3390/ijms252313125/s1.

## Abbreviations

1-NPN, N-phenyl-1-naphthylamine; AaenPBP1, *A. aeneociliella* pheromone-binding protein 1; OBPs, odorant-binding proteins; PBPs, pheromone-binding proteins.
