# Supplementary material for: Pheromone-Binding Protein 1 Performs a Dual Function for Intra- and Intersexual Signaling in a Moth
Source: Int J Mol Sci. 2024 Dec 6;25(23):13125. doi: 10.3390/ijms252313125 (PMC11642448; doi:10.3390/ijms252313125)
Supplement: Supplementary file 1 [file ijms-25-13125-s001.zip › Table S1.pdf]

Table S1 Amino acid sequences of OBPs of *A. aeneociliella* and other insects used for phylogenetic trees construction

Gene name	Sequence
>AaenPBP1	MWEKMGIKMFVVVLLGMSVSVDSSQTVVKSMTKYFFKAYEVCTKEYNIKEGTLGQIFNFWREDFTTNSRDIGCTIYCLSTKLDLLDPEGKLHHGNAAEFAMQHGSDEATAKKLVEILHTCEQTTPPNDDKCMKALDVAFCFKKELHRLDWAPDSEVLFEEIIAELG
>AaenPBP2	MCMSDKLDLVDPEGKMHHGNALEFAKKHGADDAMATQLLDLLHKCEDANPAGDDKDDCARVLEIAKCFKAEIHKLNWAPSMDLIMAEVLADV
>AaenPBP3	MMNSMLGAHLSSCMCPWKHLATSRILSQWSSFASFCFSHPCSSDIICFAVSSSRERKTSF
>AaenGOBP1	MKDITLGFGAALEHCREESGLTEEKMEEFFHFWREDFQFEHRELGCAITCMSRYFNLLTDADRIHHLNTEAFIKSFPNGEKLALQLVEVIHQCEKKFDSEQDTCWRVLHIAECFKDTCRERGIAPAMEMLLAEFIMEAER
>AaenGOBP2	MACSWMCLGLVVMAAVGSVRGTAEVMSHVTAHFGKALEECREESGLSSDIMEEFKHFWSDDFEVVHRELGCALICMSNKLSLLHGDTRVHQINMHEYVKNFPNGEVLSEKMVTLLKNCEKQYDDITDDCVRTVKMAACFKEDAKKEGIAPEIAMIESVIEIYE
>AaenOBP1	MCLLSIISVSSRHGNKRTCSKMFKEAVLIGLWLVVACRSDTPVAPPAPPQVYCGEVPSQMYNCVKLPKIIGENVTSKCGGSSSPCERVTCTFRESGFLVDGKVDKKKVSEHYDRFEAEHPEWTVAVRHVQAACLERDLPSQGLYLNCPAYDVMWCAFTSFIRSAQPSQWASTEKCAYPRQYSAACPVCPSECFAPAVPVGACNACLALPRTP
>AaenOBP2	MVRQISALLCCLCVFGISLSDSAISAESEKRCRNPPTAPQKIERVITLCQDEIKLSILREALDVIKEEHTMPAQKRRNKREVPFTHDEKRIAGCLLQCVYRKVKAVDGYGFPTLEGLVGLYSDGVNERGYFMAVLEASRECLMRHHDHFSRTVPMDNGRNCDVSFDIFECISDRIGDYCGNSGL
>AaenOBP3	MYKLCVIIIAVVCARYVDAVSEELQTLIQSKLISAGLECIKDHPLSLSDIRAFKDKRMPNNEDAKCFAACLFKNLGIMDDMGKLNPSTASESAKQVFRNNEEHRKKADQIVRVCSKVNEQMTSDGNRGCERAKLAFECLTKNAARFGFDFDFS
>AaenOBP4	MARSTILLCSLYFFALTPYLTKAMTPDQRAIIKAHFEQLGMECIKDYPITEDDISDLRGKKIPSGPGAPCFLACMMRKIGVMDDAGMLQKEDVLELAKKVFEDEEELKIISDYLHSCHQINDAAVTDGAKGCERAMLALKCMTENAPKFGIEM
>AaenOBP5	MAKFSILCLAVLAAALNVKALTQDELSEIKAAMMVHFTECNKEFGVSEETLKEAKEEKNLDKIDPCLIGCVFKRSGAVDAKGLFDTEKVLEMSKTYLKTDEDQSKFAEIVKDCAKANDEKVPDGEDGCERSKLVAICFAKHKDELVSSRR
>AaenOBP6	MFKILCLVFAVYYLQEVKSMTEMEAKLEFTKFVMKCMKEHPVEPTDLASLQQYMLPKNPEVKCLLACAYKLDGIMTQDGLYNKEHAYKIAEKMKNDDEKRLENGKKMADICAKEVNEAQVSDGEKGCDRAGHLFKCTIDNAPKFGFKLV

>AaenOBP7	MSKVGVRFVVVACILEFALALSTEQIGEIQSKFITVGEACIKDHPLSEEDIQA<br>FKYRKFPEGENAPCFSACVLKNIGFFDDKGILSRETALEHAKKIFQAGNEIK<br>TVEEFIETCSKVNDEEVTDGEKGCERAKALFSCFAENLEKFDF
>AaenOBP8	MIFKVFSVFLFVLATSECRSVSEIKQWIMKKALSCSSEVAVTSEEQQMLLA<br>HKMPNTKGAKCFTACVFKKVGWMNDKGMYDDAKAHEFATEEYANDAT<br>KLEASKKIFTICKTVNDMTDAPDGCERAYGLARCLLENAPKVGFNLDVL
>AaenOBP9	MAGLVLKIFLVSIIVNCAKAESLEDLKKQYTSTLVECAQKYQLTPGDILQL<br>QDKKMPDTESAKCMFACAYKASGMMDDHGMLSVEGLKRINEKYLSDDT<br>EKMENAFKFVDACKSVNDQKVSDGDKGCERAALIFQCSVEQAAVFNLE
>AaenOBP10	MYSLFFYFVIIISASYGDLLTQERNRGATLKPISACCDIPELGDAKPLTECSN<br>PKLLGPCNDVQCVFEKAGFLVDRNTLNKDVYKRHLRKWAEEHQGWADA<br>VERAITDCVDKDLRQYLDYPCRAYDVFTCTGIAMLKKCPKDAWKC
>AaenOBP11	MYYSWFVLFVYLSVLLHQVVPENDHENPFSSIVKKTLIATAHSCMDKVDA<br>TQADLEYLRSDPPYPEKASCIIKCLLEKVGVVKRNKYSKTGFMDAVTPLVL<br>KNKKKMAHMNTVSENCEKEINHHEVTPCQLGNEVITCIYKYA
>AaenOBP12	MCSMGYRRVVMVLAMAAAVACMDEEMAELAKMLHDNCGEETGADLSL<br>VDKVNAGADLMPDPKLKCYIKCIMETAGMMNEGEVDVEAVLALLPDDF<br>RAKNEKSLRGCGTQRGADHCDTAFLTQACWQKANKADYFLI
>AaenOBP13	MYGFLFFVLIIVSVLALDLNEYEQKCGKKLMMTKECCKSVPELPLDIKNN<br>MKECNQLPGHTCEREICMAIKMGTGTAEGKLIMDKAIENIENIFKDSKEIM<br>DAIKSECIEKDLKMYGTPDTCEIKNWKLCINVQLIKTCAEWLEDDNCKAL<br>KENSQECAQLFQ
>AaenOBP14	MTWILLLGLLGVIGGVYPATTGCKNCITLGKEEKAMFRAHSDACLPQSQV<br>DPKLVEGMLTGELADDEALKRHVYCVLLKCKVISKDGKLQKTAVLGKMA<br>TKADGKNATKVLEGCADQTGDTPEDIAWNLFRCGYDKKAVLFEYMPTNI<br>SSSDVEIL
>AaenOBP15	MKTFIVFAVCLVVALALTDEQKEKLKKHKTECLAETKVDEQLVSKLKAGD<br>YKSDNEALKKYALCMLIKSELMTKEGKFKKDVALAKVANPADKPQVEKLI<br>DTCLANKGNTPHQTAWNYVKCYHEKDPKHAIFL
>BmorGOBP1	MWKLVVVLTVNLLQGALTDVYVMKDVTLGFGQALEQCREESQLTEEKM<br>EEFFHFWNDDFKFEHRELGCAIQCMSRHFNLLTDSSRMHHENTDKFIKSFP<br>NGEILSQKMIDMIHTCEKKDSEPDHCWRILRVAECFKDACNKSGLAPSME<br>LILAEFIMESEADK
>BmorGOBP2	MFSFLILVFVASVADSVIGTAEVMSHVTAHFGKTLEECREESGLSVDILDEF<br>KHFWSDDFDVVHRELGCAIICMSNKFSLMDDDVRMHHVNMDEYIKGFPN<br>GQVLAEKMVKLIHNCEKQFDTETDDCTRVVKVAACFKKDSRKEGIAPEVA<br>MIEAVIEKY
>BmorPBP1	MSIQGQIALALMVYMAVGSVDASQEVMKNLSLNFGKALDECKKEMTLTD<br>AINEDFYNFWKEGYEIKNRETGCAIMCLSTKLNMLDPEGNLHHGNAMEF<br>AKKHGADETMAQQLIDIVHGCEKSTPANDDKCIWTLGVATCFKAEIHKLN<br>WAPSMDVAVGEILAEV
>BmorPBP2	MKLQVVLVVLTVEMVCGSRDVMTNLSIQFAKPLEACKKEMGLTETVLKD<br>FYNFWIEDYEFTDRNTGCAILCMSKKLELMDGDYNLHHGKAHEFARKHG<br>ADETMAKQLVDLIHGCSQSVATMPDECERTLKVAKCFIAEIHKLKWAPDV

	ELLMAEVLNEVSWKS
>BmorOBP5	MKQRLRVLLLRFCILQTVLSESGVDVVKNLSLSFARFFLECDEERHFQPEV
	RLKVMTFWYSESSTWDRDVGCAFLCIFKKMEIDNPQDPSYRTHLELLSFA
	NSEDNKIANQMVEIFYACGENTETDPCLWALEQVKCYKNRINQLGLTPTF
>BmorPBP3	MARYNIVVAVLVLGVVGARGSSEAMRHIATGFIRVLDECKQELGLTDHILT
	DMYHFWKLDYSMMTRETGCAIICMSKKLDLIDGDGKLHHGNAQAYALK
	HGAATEVAAKLVEVIHGCEKLHESIDDQCSRVLEVAKCFRTGVHELHWAP
	KLDVIVGEVMTEI
>BmorOBP7	AVTEEELKIEFTKLVMKCTKDHPVDMSELMQLQQLIAPKKTESKCLLACA
	YKLNGVMTSQGLYNLEHAYKIAEMSKNGDEKRLENGKKVADICVKVND
	VEVSDGEKGCERAALIFKCTLENAPKVFKFGSSEYNCQ
>BmorOBP8	MLRVVVICVCFLVIAPYGINASSLDDLKMVYKNVIKECVGDYPITAADLKL
	IKARQIPNDDIKCVFACAYKKTGMMTEEGMLSVEGIKDMSQKYLSDNPEQ
	LRKSKEFAEACSSVNDQQVSDGTKGCERAALIFKCSTEKITNFGFEL
>BmorOBP9	MLRVVVICVCFLVVAPYGINAVSYEQKIKIRDQLDRAGFECFKDHKITEDDI
	KNLRANKPATGENVPCFIACVMKKTGVMNDQGVIRKGPVLELAKKVLAD
	DKDIKKLQDYIHSCSHVNSETVHDKGKGCEFAMQAYTCMSANASKFGFNI
>BmorOBP10	MLRVVVICVCFLVIAPYGINAVSDEQKIKIREQIDKSGFECFKDHKITEDDIK
	NLRARKPATGENVPCFIACVMKKTGVMNDQGVIHTEPVLQLAKKVLTDD
	KDIKKLQDYIHSCSHVNSKTVHDKGQGCEFAIQTYTCMSANASKFGFDV
>BmorOBP11	MSANSFVVLAFCALAVGVNALTEEQKAEITKSSLPLIAECSKEFSVNQGDI
	DAAKKLGDPSGLNSCFVGCFMKKAGIINASGLFDVAATIEKSKKYLTSEED
	LKAFEKLTETCAPENDKPVSDSDKGCERAKLLLDCFVANKGSFSVFSL
>BmorOBP12	MTSFMVFFVLSVLTLKYSDALTDEQKNKIQSKFIEIGAECIVEHPISIDDINS
	FKNKKFPSGVNAGCFVACIFNKIGLFDDKGNLSHNSALEKAKGIFNADEEV
	KNLEEFLNRCAKVNGEAVGDGVKGCERAKLAYNCLIENSLEFGFNIDF
>BmorOBP13	MLKIHVLLCFGMAILYFGSAKAVTPEESKAFEAFAKPVIEQCQKDFGMDK
	ESFAQKNLDEIDECLIACVVEKFGITNDEKIDGDALKALVTKFVGNEEERN
	KINKIVEECTEDANKSGDGTCNTSTILFLCLLKNGKDLWGF
>BmorOBP14	MERKDFYLLIVVVALTSGVSSMSRQQLKNSGKMLKKQCMGKNDVTEEEI
	GDIEKGKFIEQKNVMCYIACIYQMTQIIKNNKISYEASIKQIDLMYPPELKE
	SAKASAGRCKDVSKKYKDICEASYWTAKCMYEDNPKDFIFA
>BmorOBP15	MFLKNIFIECVLLYFVMLNTSFVNTMTKQQIKNSGKILKKACISKNDVTED
	QISDIDKGKFIEDKNVMCYIACVYSMSQVVKNNKFVHDAMVKQVDMMF
	PTEMRDAVKASIANCRGVAKNYKDICEASFWTAKCMYEFDPANFVFA
>BmorOBP16	MRISFLFLISVTIITFDSVFAMTRAQVKKTMTIMKNQCMPKNGVTEDQVG
	KIEEGIFLENHNVMCYIACVYKTIQVVKNDRLDKDLISKQIDVLYPQEIRES
	TKKAVGDCINLQEKYDDWCEGIFRSTKCLYEKDPANFIFP
>BmorOBP17	MTRQQLKNSGKIMKKTCMPKNDVTEEEIGQIEQGKFLEQRNVMCYIACIY
	TVTQVVKNNKLSYDAVIKQVDVMFPAEMRPAVKAAAENCKDISKTFKDIC
	EASYWTAKCMYDFDPKNFVFP
>BmorOBP18	MILIVIAKFLILISLCETMTMKQIKNTGKMMRKSCQPKNNVDDEKINPIND
	GVFIEENEVKCYIACIMKMANTMKNGKLNFEAAMKQADLLLPDEMKEPT
	KEAIVACRKVADSYKDVCDASFHVTKCIYNHNPSVFFFP

>BmorOBP19	MTSAKTDVEIKAWFLGQAVECSKDHPVTTEELRMHKHELPDSKNAKCLM<br>KCVFRKCNWLDSKGMYDINAAYASSTKDFSDDKTKQENANKLFDTCKSV<br>NEENVGDGEEGCDRSLLLAKCLTKAAPQVSIYYS
>BmorOBP20	MAVHIFLILASYMALAAHGQLDDEIAELAAMVRENCADESSVDLNLVEKV<br>NAGTDLATITDGKLKCYIKCTMETAGMMSDGVVDVEAVLSLLPDSLKTK<br>NEASLKKCDTQKGSDDCDTAYLTQICWQAANKADYFLI
>BmorOBP21	MITASLHVIFALLAFVYGGKDKPVLSEEIKEIIQTVHDECVGKTGVSEEDIT<br>NCESGIFKEDVKLKCYMFCLLEEAGLVNDDGTVDYEMFTSLIPEEYFDRA<br>TKMIFSCKELDTPDKDKCERAFEVHKCSYEKDPDFYFLF
>BmorOBP32	MYSHKYLNDFTNIPEILIILLSSVALMSYGYNTKLFSHSLGSEPSLSILYARD<br>KKSDKVTNECLMEMYPKNLYKYPLRIDRNDIPCIIHCVLKKFGIISNDGFIN<br>IKNYYRRVQAIHRYDPRILISDVGETCAQNINGMNLDHDVCKKAKVFNDC<br>TQLYAISYREPEDW
>BmorOBP33	MYAHDKLSDMIADQCLNEMYPRSKRLEIEESDEPCIIFCVLKKFGIMSPTG<br>VINLEAYRKRVQLPEQLAQRNSINDFGSACLESAEATQHKQDVCKKAKVF<br>NECTHLYKILLK
>BmorOBP34	MEKMILLNVFAVVLPCVLASRTRGSSGTLVDFTDPKVQGHLDALVRMAQS<br>CVIKVRATPKDVRAYFTNSSPVSRSGQCFATCMLEQSDIINHGKVNRDLLV<br>HLAGLVNGKNSRVVRKLNSVSRLCLDSISGMTDRCQLASTYNDCLNENMI<br>EFAFPLDIAEEAVRKMPFHLIQPK
>BmorOBP35	GMSTHVLDFKRNMTECLKEVQNNDKRPIKRLSPKQESPIHGECLIACVLK<br>KNGVIQNGKVNKDNLMALVSKFHAKETKLMKKLEKNLDRCINISVKNHD<br>ECSLASQLNDCTNDIMASSKQKILFNY
>BmorOBP36	MAVSEISRILTFLTIVSFIYIVYSFKPLTKDEHIERYNKMNEDIEPFRKNLTEC<br>ARQVKASMADVEKFLKRIPQSNMEGKCFVACILKRNSLIKNNKLSQENLL<br>EVNRAVYGDDSEVMSRLKTAILECSKIVEDIFEICEYASVFNDCMHMKME<br>HILDKITMERRMEALGQMSSNPDEWSEEEDEMLKLVKDEL
>BmorOBP37	MFYPFRFTLLFYGLFVIYLVRAEPEKENHFTLALKKTLFSTARSCMSHVNA<br>NETDLEYLRKDPPFPDKAACIIKCLLEKIGVVKNNKYSKMGFLTAVSPLVF<br>TNKKKLDHYKSVSENCEKEINHDQTTECELGNEVVSCIFKYAPELHFKT
>BmorOBP38	MANLVLLLTFVLMTLSMARLKSTEAPKSKTALFNDQDNMGYEELDMEEI<br>MSACNESFRIEYAYLESLNDSGSFPDETDKTPKCYIRCVLEKTEILSENGVL<br>NPATAALVFAGERNGKPMSDLEEMAVACADRHEKCKCEKAYNFVKCLMY<br>MEIDKYEKKN
>BmorOBP39	MVRKISALLCCFCVLGISMCDSAISTDNEQRCKNPPTAPQKIERVITLCQDE<br>IKLSILREALDVIKEEHTMPAERKRNKREVPFTHDEKRIAGCLLQCVYRKV<br>KAVDGFGFPTLEGLVGLYSDGVNERGYFMAVLEASRECLMKNHDKFSRTT<br>PMDNGRNCDVSFDIFECISDRIGEYCGTSGL
>BmorOBP22	MLKVFVVVVCTLGASQLCAALYTQKVAVSFPKDKTTIVVEAMKSCIAKTG<br>ANPNVIEVISSGKVSEDEKFKEFFYCACNDIGVVNPDGHIKVKECIELFPKE<br>TQPLVEPVIKNCDKEGVNKYDTLFKYLKCFQETSPVRVTLA
>BmorOBP23	MTSKVLLSCVVLAVLATTVLAEDSRKLVSFAPEVAKKLKVLIQECLNENGL<br>GEDAIEVIRAGEYREDEPFQNLVYCAYKKFGALDENNRIISQVAAASFPKDI<br>DVVTVIESCGKEDGNTPVEQVFKYFKCFQKNSPVRMQLY

>BmorOBP25	MKSVVLICLAFAVFNCGADNVHLNEDEREKANWYTAECGVETGVSTEVINAAKIGKYSKDKAFKKFVLCFFKKSAILNSDGTLNMVVALAKLPSGVNKSEAQSVLEQCKNKTGQDAADKAFAILQCFHKGTKTHILF
>BmorOBP26	MKSVVLICLAFAVFNCGADNVHLAETQKEKAKQYTSECVRESGVSTEAINAAKIGKYSKDKAFKNFVLCFFNKSAIFNSDGTLNMDVALAKLPPGVNKSEAQSVLKQCKNKTGQGAADKAFEIFRCYYKGTKTHILF
>BmorOBP27	MKSVVLICLAFAVFNCGADNVHLTETQKEKAKQYTSECVKESGVSTEVINAAKTGQYSEDKAFKKFVLCFFNKSAILNSDGTLNMDVALAKLPPGVNKSEAQSVLEQCKDKTGQDAADKAFEIFQCYYKGTKTHILF
>BmorOBP28	MLKVFIVTFFAFQLSAIARLQANGCVAVPFPKDKTIIIVEAMKSCIAKTGANPNFIDVIRSGKVSEDEKFKEFYYCTCNDTGFVNPDGHIKVKECIELFPKETQPLVEPVIKNCDKEEGVNKYDTLFKFLKCFQETSPVRVALA
>BmorOBP29	MTGPAAAAVLLALLAAAGQATTGCKNCVILGKEERAMFRSHSDACLAQSRVEPRLLESMMNGELIDDAALRKHVYCVLLSCKMIGKDGKLLKAAILGKLAARPAGRDVTKVLEACAEQPGASPEDVAWNIFRCGYNRKAVLFDYMPAGGASSGNTENHP
>BmorOBP30	MRSFVILLNYGLLCCGQFMAEDYYYDIVTRDPDDLMREKENEVRALRAFQADCAEDVQVKPDLVVNLKSGDWQTEDVSLKKWALCVLMKLGLMTAQGVFKMNEAMSKIPDMNDKIIAEKLIDDCLSLQATTPHDAAWNYIKCHHQKDPEGNFSSLNIF
>BmorOBP31	MKTFIVFVVCVVLAQALTDEQKENLKKHRADCLSETKADEQLVNKLKTGDFKTENEPLKKYALCMLIKSQLMTKDGKFKKDVALAKVPNAEDKLKVEKLIDACLANKGNSPHQTAWNYVKCYHEKDPKHALFL
>BmorOBP40	MSEFIQPSWRTQCNFRLNWDNRNRLSIDISHGAATTQTPVPTTKPKALRDFMVVPQSCDKTTCVFKKLNIVSDKGVVDVKSFIKLLDKFTNSYPVWNSAKARVITTCLRKSLIAYDGGCELNNILACTFDVLSENCPLNGNNQTC
>BmorOBP41	MLTILFLLPIVVGVLSGNIPEQPRVYCGELPNTIYSCLGNPKIIQPEVSEKCNKPISECDKTRCIFKESGWAKNNVIDKKKVSDYFEQFAKDNPDWSAAVQNFKTTCLSDSLKPQGVDTNCPAYDIIHCALISFIKFASPSQWSTSEQCVYPRQYAGACPVCPERCFAPSVPNGSCNACLALLRTP
>BmorOBP42	MMGYACVFVILAVLQAISAEDPPGLPPFLKDAPEKCKSPPRVKNPNECCISEPFFKEADFIECGIEKPGSERGPPDCSKQNCLLKKYNLLKNDETPDIEAIKSLLDKYIEKNPSFKSSVEKAKECLREDLPGPPQICLANRMTLCIGTVLLMECPDEKWNTTDDCKAFKDHMTECQKYFPK
>BmorOBP43	MKVCVLFAIFTVAQAAKATLKPISACCNIPELGNPEPLAECSNPKLPGPCKDIQCVFEKSGFLTENKTLIKEAYKTHLRQWAKEHEGWSVAVEKAISDCVDKDLRQYLEFPCSAYDVFTCTGIAMLKKCPNEHWTC
>BmorOBP44	MSRLVLFFTILVVLQEFIINLYFNFITEIDSCCVKKYPKLFDSEFITECYNTQRKANDKCERDMCVARKLNLLTEEDSINKDALLRFVEEGFKTEIDLVNAIKKKCFEEDISNIGKPEMCEVAKYKICITSRMAEDCPKWDSKGICSSAQQKVENFMKMLS
>SlitGOBP1	MLLLLALPLLAAVLPLRADVNVMKDVTLGFGQALDKCRQESQLTEEKMEEFFHFWRDDFKFEHRELGCAIQCMSRHFNLLTDSSRMHHENTEQFIQSFPNGEVLARQMVELIHACEKQHDHEDDHCWRILHVAECFKQACVQRGIAPSM

	EMMITEFIMEAEAR*
>SlitGOBP2	MATVTSSVMGTAEVMSHVTAHFGKALEECREESGLSAEVLEEFQHFWRD<br>DFEVVHRELGCAIICMSNKFSLLQDDSRMHHVNMHDYVKSFPNGHVLSE<br>KLVGLIHNCEKQFDSMTDDCERVVKVAACFKVDAKAAGIAPEVAMIEAV<br>MEKY
>SlitPBP1	MANARWRFVFVVYALYLTSAVLGSQDLMAKMTKGFTRVVDDCKTELNV<br>GDHIMQDMYNYWREDYQLINRDMGCMLLCMAKKLDLMDDQTMHHGK<br>TEDFAKSHGADDDVAKKLVSVIHECEQQHTGIADDCMRVLEVAKCFRTKI<br>HELKWAPSMEVIMEEVMTAV
>SlitPBP2	MSLRVALVVAASLLVVVQASQDVMKNLAINFAKPLDDCKKEMDLPDSVT<br>TDFYNFWKEGYELTNRQTGCAILCLSSKLEILDQELNLHHGRAQEFAMKH<br>GADEAMAKQIVDMIHTCAQSTPDVAADPCMKTLNVAKCFKLKVHELNW<br>APSVELIVGEVLAEV
>SlitPBP3	MGSRNVFVALVVLTVAMRETEPSKDPMKYIASGFVKVLEECKHELNMND<br>HLIADLFHYWKLEYTLLNRDTGCAIICMGKKLDLLDANGRMHHGNAQEF<br>AKKHGAGDEVASQIVQIIHECEKKHERDDDECLRVLEVAKCFRTGIHELN<br>WQPNVEVIVSEVLTEI
>SlitOBP5	MTKVLFAIVLTMVTFAVVLSASTKEAMTTTMSDQVNSIDVDVLAVMDMC<br>NDSYRIDPTYLQALNESGSFIDETDKTPKCFIRCVFENVGIVSEDGKQFNPA<br>RAAVIFAGERNGKPMEDIADMTALCATDRQETCPCDRSYKFLRCLMSMEI<br>ERYEKS
>SlitOBP6	ESKFGEIVKRTVIATAHTCMDHVNATAKDLEHLRDEPPYPETSACIVKCLL<br>EKVKYMRKQTN
>SlitOBP7	MFTEALPLFVILVAVTHGGKNKPVFSDEIKEIIQTVHDECVAKTGVAEEDIT<br>NCENGIFKEDAKLKCYMFCLLEEASLVDDDDTVDYDMLVSLIPDEYYERT<br>TKMIFACKHLDTPDKDRCQRAFEVHKCSYEKDPDLYFLF
>SlitOBP11	MSKFTCLVLCVVAVSLNGVHATAEEKAAFIEAVKPYVQECSKEHGVTPEDI<br>KSAKAAGNADGINSCFLSCVYKKAEVINEKGEYDVDKALEKLKKFVSNE<br>DDYAKFANIGKKCASVNEKSVSDGEAGCERAALLTSCFLEHKSEISA
>SlitOBP12	MSVVRCSSFLVALFCFVSVNAMSGDEEAGIKEALRPFVQECADEFGITEEQ<br>FEEAKKKASAADIDPCFMSCFLKKAEFFDSQGKFDVDSTMAFAKEHLTSE<br>PAMKFVEAVGDECVKINDEDVSDGDKGCDRAKLLFECIAETKKKME
>SlitOBP14	MDQKRICLFVIAMFLASGSDAMSRQQLKNSGKMLKKNCMNKIGVTDDQ<br>VGSIDKGKFIEDRKVMCYIACIYELTNVIKNNKLNYEASIKQIDLMYPPDV<br>KESAKAAVEKCKDVQKKYKDICEASFYAAKCMYEYKPEDFIFA
>SlitOBP15	MFNNCFVYSMTREQIKNSGKLIKKTCSAKNDLTEDEVKDVDKGKFIEKKD<br>FMCYIACVYKMGQTVKGSTINHDMMLRQVDMMFPNDMKAPVKAAIEH<br>CRPVAKNYKDLCEASYWTAKCIYDFDPANFMFP
>SlitOBP17	MRTFRLLCCILSIFFIFDQSYGMTRQQLKNSGKLMKKSCMPKNDVTEDEV<br>GDIEKGKFIETRNVMCYIACVYTMSQVVKNNKLSYEAVIKQVDVMFPAE<br>MRDAVKAAATHCKETTKKYKDLCESSYWTAKCMYDYDAQNFVFP
>SlitOBP18	MILXYTQKLTNMLLTKIVKFFILVATCEAMTMKQIKNTGKMMRKTCQPKN<br>NVEDEKIDPLSDGVFIDEKEVKCYMACIMKMANTIKNGKLNYDAAMKQA<br>DLLFPDDIKEPAKEAITACRKVADAHKDICDASFHVTKCIYNHNPSIFYFP

>SlitOBP20	MWVQALVLTLATLVTLVAAAVEMDEDMAELARMVRDNCAGETGVDVAL VEQVNAGAELMPDDKLKCYIKCTMETAGMMADGEVDIEAVLALLPPSLA EHNAPALRACGTQRGADHCDTAFRTQQCWQNANKADYFLI
>SlitOBP21	IHYLCAARAVHKIKNITNCENGIFKEDAKLKCYMFCLLEEASLVDDDDTV DYDMLVSLIPDEYYERTTKMIFACKHLDTPDKDRCQRAFEVHKCSYEKDP DLYFLILRREQLASRDDCVAISGIN
>SlitOBP3	MKSFVVICIVFVVGVCATEKGNKIASECIKESGVKSDVLAEAKKGNLGDD PAFKEFTYCFFKKVGIVGEDGKLNRDVAIAKLPSGVDKAEAEKLLDSCKS KTGKDAVETVYEIFKCYQHGTKSHIMFAS
>SlitOBP4	MKTLLVFAACILVAQALTDEQKEKLKKHRTECLTETKVDEQLVNKLKGGD YKMDNEALKKYALCMMMKSELMTKDGKFKKDVALAKVPNPADKPTVE KLIDACLANKGNTPHQTAWNYVKCYHEKDPKHAIFL
>SlitOBP1	MFKLCVFLALGFVACHGASNSNPGTPNANPGTYCGVTPDNIYRCLNNPRV VTPEVSTKCGSQFTECEKMTCIFRELKWSKRGAIDKAKVRAYFDQYETEH PEWAQAVQHVKAFCLASELRAQGVFLNCPAYDIMQCVLASFIKHASPSVW STATDCAYPKAYAADCPVCPSDCYSPQIPYGSCNACYTQPRTV
>SlitOBP2	MVRKISGLLCCLCVFGISFSDSAISADSESRCRNPPTAPQKIERVITLCQDEI KLSILREALDVIKEEHTMPAQRRRDKREVPFTHDEKRIAGCLLQCVYRKV KAVDGYGFPTLEGLVGLYSDGVNERGYFMAVLEASRECLMKNHDKFSRT VPMDNGRNCDISFDIFECISDRIGEYCGTSGL
>SlitOBP13	MITSSSLLVLTAVVQVLFAQQPVFESGPPEPWGPPQRPAHRRQFLPRIPKRC WVPPQRINVYNCCPIPTLYPDEDMQSCGFEKTSGNTDQPQKPVFRPEGTC KEGYCVMGKFDLLFANNSVDFVKFREYLDNWAESYPEFANAIRIAKQECA QDGGPEVPPICEPDKLFLCLTSTIFWNCKLRDGDGCAALQEHMNECKQYY TRQMEPTMKDIEVR
>SlitOBP16	MLAEELKDCFDGSGPKDPMKCEIDLCIAKKKGFATDDGKLDIKKFEEVITK DVGSDKDLLDEIKTNCINGDLNNYGPPEFCDFIKIKHCVTLHMMNHCSEW SDDGNCKVVKELVGKCAKVI
>SlitOBP19	MYSKICILLFISYTCLVTADSVSFIKKCKWDDGKCAKESGQNVIQKFAAGIS EYNVGVSDPLHIEYVDASSPNMKLIVTDVVVTGLRNCEVKKIQRFEDSSK LIVKLLCAAELNGKYDMKGQLFVIPIEGNGGLYSKVPKIQINAEVDLNTKQ GKDGKDHWIVKSWRHTFELKDKSTVKFENLFPDNEFLRTSTNELIAQNGN DVIIEIGANLIKAIVGKIVENIKKFFIAVPIEDLSL
>SlitOBP10	MKEGNRYSHERRITNDSGDQLMVINATDDDYSGYGSGNMGEKLLTSVPR PATPSNNINKNNINRTKRNEPLLNRPDSDQCLSQCVFANLQVVDSKGIPRE AELWNKVQSSVTSQQSRSALHDQIQACFQELQSEAEDNGCSYSNKLERCL MLRFSDRKVDGKGNAKKSSTEQTG
>HvirGOBP1	MPGVLRALLLLAAAAPLLADVNVMKDVTLGFGQALDKCREESQLTEEK MEEFFHFWRDDFKFEHRELGCAIQCMSRHFNLLTDSSRMHHDNTEKFIQS FPNGEVLARQMVELIHSCEKQFDHEEDHCWRISHLADCFKSSCVQRGIAPS MELMMTEFIMEAEAR
>HvirGOBP2	MTSKSCLLLVAMVTLTTSVMGTAEVMSHVTAHFGKALEECREESGLSAEV LEEFQHFWREDFEVVHRELGCAIICMSNKFSLLQDDSRMHHVNMHDYVK SFPNGHVLSEKLVELIHNCEKKYDTMTDDCDRVVKVAACFKVDAKAAGI

	APEVTMIEAVMEKY
>HvirPBP1	MMSVRLMLVVAVWLCLRVDASQDVMKNLSMNFAKPLEDCKKEMDLPDS VTTDFYNFWKEGYEFTNRHTGCAILCLSSKLELLDQEMKLHHGKAQEFA KKHGADDAMAKQLVDMIHGCSQSTPDATDDPCMKALNVAKCFKAKIHEL NWAPSMELVVGEVLAEV
>HvirPBP2	PKWVFARAFCLVLMMGSAMSSKELLTKMTGGFTKVVDHCKTELNVGDHI MQDMYNFWREEYQLVNRDLGCMIMCMTAKLDLVGDDQKMHHGKAEEF AKSHGADDALAKQLVGLIHGCETQHQAIEDHCSRTLEVAKCFRTKIHELK WAPSMEVIMEEIMTAA
>HarmPBP1	MEFHRSTMMSVRLALVVAVCLFIRVDASQDVIKNLSMNFAKPLEDCKKEM DLPDSVTTDFYNFWKEGYEFTNRQTGCAILCLSSKLELLDQELKLHHGKA QEFAKKHGADDAMAKQLVDLIHGCAQSTPDVADDPCMKTLNVAKCFKA KIHELNWAPSMELVVGEVLAEV
>HarmPBP2	MAASRWLFARAFCLVLMMGSAMSSKELLTKMTGGFTKVVDACKTELSV GDHIMQDMYNFWREEYQLVNRDLGCMIMCMTAKLDLIGDDQKMHHGK AEEFAKSHGADDALAKQLVGLIHGCETQHQAIEDHCSRALEIAKCFRTKIH ELKWAPSMEVIMEEIMTAA
>HarmPBP3	MGSRHVFFALVVLAVSVRKAEPSKDAMQYITSGFVKVLEECKHELNLNEQ ILADLFHFWKLEYSLLGRDTGCAIICMSKKLDLLDANGRMHHGNAAEFA KKHGAGDEVASKIVTIIHECEKKHEQDGDECLRVLEVAKCFRTGIHELNW QPKVEVIVSEVLTEI
>HarmGOBP1	MPGVLRALLVLAAAAPLLADINVMKDVTLGFGQALDKCREESQLTEEKM EEFFHFWRDDFKFEHRELGCAIQCMSRHFNLLTDSSRMHHDNTEKFIQSFP NGEVLARQMVELIHSCEKQFDHEDDHCWRILHVAECFKGSCVQRGIAPSM ELMMTEFIMEAEAR
>HarmGOBP2	MTSKSCLLLVAMATLTASVMGTAEVMSHVTAHFGKALEECREESGLSAEV LEEFQHFWREDFEVVHRELGCAIICMSNKFSLLQDDSRMHHVNMHDYVK SFPNGHVLSEKLVELIHNCEKKYDTMTDDCDRVVKVAACFKVDAKAAGI APEVAMIEAVMEKY
>HarmOBP1	MSKFTFFVLCVVAVSLSKVYASDEDKAKLHEALKPLVEECMKDHEVSLDD LKAAKEAKSADGVKPCFLACVYKKAEVLNDKGEFDADHALEKLKEFVS DEDVLAKVAEVGNTCKAVNDKAVSDGDAGCERAALLTACFLEHKAEILV
>HarmOBP2	MMDRKRLCLLIIAMFLAQGSDAMSRQQLKNSGKMLKKNCMNKNQVTED QIGSIDKGKFVEDKKVMCYIACIFEMTNVVKNNKLNYDASIKQIDLMYPP DLKESAKAAVEKCKDVQKKYKDICEASYWTAKCMYDFKPEDFIFA
>HarmOBP3	MSKFTCFVLCVLAVSLGEVRSNALEKAAIRAAVYPLIVDCAKEHAVTLEQL KAAKASHSAEGINPCFQSCVYKKTGIFNDNGEYDVANAKTKLQKFVTDE DEYARIAEVGKTCASVNDKSVSDGAAGCERAALLTACFLEHRAQIII
>HarmOBP4	MSKLTCVVFAAVAVVFSNVNADDETRASFRQVLGPLVMECRNEFGITEDD LKKAQQERSPDALKPCFIACVFKKFGIITSAGKYDSDASISRIKDVVKNDD LLAKLKSVGEKCNSVNDASVSDGDAGCERAALLAKCFIENKSELSI
>HarmOBP5	MSKFTCLVLCVVAASLSQAYASEEEKAAFREAIKPIVEECSKEHGVSHDEL KSAKDNQNADNIKPCFLGCVYKKAEVFNSKGEYDVDKALEKLKKFVSND EAYAKFAEVGKKCASVNDKAVSDGDAGCERGALLTACFLEHKAEVPL

>HarmOBP6	MSKFTCLLLCVVAVSLSKVHATEEEKEAIRAAVRPIMQECGKEHGVTLDDL KAAKAAHSADGIKPCFQSCVYKKAGIFNDNGEYDIANAKTKLQKFVTND EEYARIAEVGKMCASVNDKPVTDGAAGCDRAALLTACFLEHRAQIII
>HarmOBP7	MFRFGVLSFVVLLFCMESSYALSSEEELSIKEALHPFVVECAEEYGMTEEM FEEAKKKGSAEDIDPCFMSCFLKKTGFFDDSGKFDAEKSISFAKEHITSESA IKFLEAGAGECVKINDEDVSDGENGCDRAKLLFDCLTELKKKMSE
>HarmOBP7.2	MSRFGVLSFVVLVFCMENIYALSSEEELSIKEALHPFVVECAEEYGMTEEM FEEAKKKGSAEDIDPCFMSCFLKKTGFFDDAGKFDAEKSISFAKEHITSETA IKFLEAGAGECVKINDEDVSDGDKGCDRAKLLFDCLTDLKKKMSE
>HarmOBP8	MLLIEIVKFLTLVAMCEAMTMKQIRNTGKMMRKSCQPKNNVADEQIDPIA EGVFNEDKEVKCYMACIMKMANTIKNGKLNYEAAIKQADLLLPDDIKEP AKEAITACRKVADAYKDICDASFHITKCIYTQNPGIFYFP
>HarmOBP9	MCKFSVLFLYSAVMAVNIWSASCISEEDKAAIITAIAPLAQNCGSECGLDND DFEKYKEDGSDMDPCFKACLMTQMGVLDKEGKYDGKGLHKAMEEADY PGDKDDAQKFLDELDRCFDAKGDNSGSDEEAKMKRADVLFRCMQDMKE K
>HarmOBP9.2	MCKCSVVFLYLAVMAINIWRASCLSEEDKAAIITAIAPLAQNCGSECGLDN DDFEKYKEDGSDMDPCFKACLMTQMGVLDKEGKYDGKGLHKAMEEAD YPGDKDDAQKFLDELDRCFDAKGDNSGSDEEAKMKRADVLFQCMQDM KEN
>HarmOBP13	MFTGTLPLVVFLATFAYGGKEKPVFSDEIKEIIQTVHDECVAKTGVAEEDIT NCENGIFKEDPKLKCYMFCLMEEASLVDDDDAVDYDMLVSLIPEEYVDRT TKMIFSCKHLDTPDKDKCQRAFEVHKCSYEKDPDLYFLF
>HarmOBP18	MKSFVVFCVLVAGAFAANVSLPPKQNEKANQIATECMKESGLKPEVLAEA KKGHISDDEHLKKFTFCFFKKAGIVSEDGKLNTEVALAKLPPGVDKAEAE KLLETCKGKTGKDVTDTVFEIFKCYHHGTKTHILLGF
>HarmOBP16	MFKLCVVLAFIVATCHGGTLERTSSTCGQIPRELTACLDLQPAVSPEIQEKC RRANECERLTCVFREYNLLDGAEVNKERTAAFLDNFVKQYPSWEVAIDVA KTSCLRSSGLKPQGVFLDCPAYDIIQCVFANLVKNALPSQWSSMSQCNHAR EFAAACPICPDACFAPLVPIGTCNACSAARRSS
>HarmOBP17	MRAWSVTLVALLGALGAARAVAMDEDMAELARMVRENCAAETGADVAL VERVNAGADLMPDDKLKCYIKCTMETAGMMADGEVDIEAVLALLPPELA EHNAPSLRACGTVRGADHCDTAFRTQQCWQNANKADYFLI
>HarmOBP18a	MTRQQLKNSGKLMKKSCMPKNDVTEEEVGDIEKGKFIESRNVMCYVACI YTMTQVVKNNKLSYEAVIKQVDMMFPAEMRDAVKAAATSCKDITKKSKD LCESAYWTAKCMYDYDAENFVFP
>HarmOBP22	MTREQIKNSGKLIKKTCMAKNDLSEDQVKDVDKGKFIEEKPFMCYIACVY KMGQTIKGNTVNHDMMIKQVEMMFPNEMKAPMKAAIEHCRPVVKKYK DVCEVSYWTAKCIYEFDPPNFMFP
>MsexGOBP1	MGQNTRSLVLVVLVGLVGAVSADVQVMKDVTLGFGQALEQCREESQLTE EKMEEFFHFWREDFKFEHRELGCALQCMSRHFNLLTDSSRMHHENTDKFI KSFPNGAVLSKTMVELIHNCELQHDAEEDHCWRILRVAECFKISCTKAGIA PSMEVMMAEFIMETENK
>MsexGOBP2	MVNRLILVVVVVFITDSVMGTAEVMSHVTAHFGKALEECREESGLPVEV

	MDEFKHFWREDFEVVHRELGCAIICMSNKFELLQDDTRIHHVNMHDYIKS<br>FPNGQVLSEKMVQLIHNCEKQYDDIADDCDRVVKVAACFKKDAKKEGIA<br>PEVAMIEAVIEKY
>MsexPBP1	MKVAVVAIVVYLAVGNVDSSPDVMKNLCLNFGKALDECKAEMNLSDSIK<br>DDFANFWVEGYEVSNRDTGCAILCLSKKLDMIDPDGKLHHGNAMEFAKK<br>HGADEAMAKQLLDIVHNCENSTPPNDDACLKTLDIAKCFKKEIHKLNWAP<br>NMDLVVGEVLAEV
>MsexPBP4	MKEAGVRFKILLFLIFPVVTGNFKGKQIMRSVAETFGRTVFECQNEVLMKF<br>GSGILNDIFRYWHEGQPLEDRDLGCIFRCILLKLELVNDNGRLIDANADGF<br>FQANGADESMTKHLIELYHSCYQTMRFPQDDCMLILEIGKCCREGVRNAH<br>WTPGSK
>MsexPBP3	MAVIPIFTVLLMMTAVKEIAPSSDAMRHIANGFLKVLDQCKHELGLTDQIV<br>VDLYQFWKLQYALLNRDTGCAIICMSKKLDLLDGTGRMHHGNTQEFAVS<br>HGATDEVASKVVVIIRDCEKQQEGEQDDCVRVLEVAKCFRTAIHELNWAP<br>NMEVVVDELLTEI
>MsexPBP2	MVSTKWCFLLVTIAVLTMEVVSASQEVLKQMSVGFSKVLQTCKTELSVG<br>DHIIQDFYNYWREDYDLLNRDFGCMVICMAVKHDLINDQLTMHHGNAHA<br>FAKTHGADDDTAQQLVTILRECEAKHQSVEDVCNRALEMAKCFRTKIHEL<br>KWAPAMEVVLEEIMTSV
>MsexOBP8	MCKPMYCVVFSIIYLSIVAAQKADNGNTKIANLQSNDQDSMDNVDVEDI<br>MNQCNETFRIEMAYLQALNESGSFPDETDRTPKCFLLCVLDNTGVMMKD<br>GDFDPERTAVLFAGERAGKVMDGIQDMAAACADRKEKCKCEKSYNYLK<br>CLMTMEIEKYANNN
>MsexOBP11	MWKISYFLFVFGVAANLKHAYAVTEEERLMLDSLMKPKVLACVEEFGLK<br>DFSIEDIRKDHEIDPCLLQCFLKKAEVFVDGMINLEKADETLREVINDEDE<br>VEQIMEKGKECADEANGSDVSGDDEDCARVAIFHSCLREKNGLFMATS
>MsexOBP12	MPKHDVTEDQVGGIEQGKFLENRNVMCYIACVYSMSQAMSKQQLKNSG<br>KMFKKQCMGKNKVTEDEIGEIDKGRFVEQQNVMCYIACIYQMSQVVKN<br>NKLNYEASLKQIDIMYPPELKDTAKGALEACKDIAKKNKDLCEASFKTAK<br>CMYEYSPKDFLFP
>MsexOBP13	MLFLVVAKFFILLSLGEAMTMKQIRNTGKMMRKQCQPKNNVEDEKIDPL<br>GKGVFINEKEVKCYMACIMKMANTIKNGKINYEAAMKQADLLLPEDIKE<br>PAKEALTSCRKVADSHKDVCDAAFYISKCIYEYNPDIFYF
>MsexOBP14	GITEKQRLHIREELIEIGTECIIKNPITIGDVRSFRNKQFPNGPNAGCFIACVF<br>NKAGIMLGLLEINNFPTVQTQDALLRAYLIKQELFDDEGLVSQKTATEKAT<br>KVFDDETELKNYEQFIAVCDKVNEESVSDGQKGCERAKLAFQCLIQNSKQ<br>VFIPYNLLLGSR
>MsexOBP15	MRRKLLNIKENPFTSVVRKALINTARSCMVHVNATQEDLEYLRKDPPFPE<br>KASCIVKCLLEKIGVVKNNKYSKSGFMMVVTPLVFANKKKLDHMKTVSE<br>NCESEIVHKESSPCDAGNEVTTCIFKYAPELHLRG
>MsexOBP23	MYAHDKLSDMVAEQCLNEMYPKSKRIEIQESDEPCIIFCVLKKFGIMSTNG<br>VINLEVFRKRVQNSHQHEQRNTMNDIGSNCLESAEATQHKQDVCKKAKV<br>FNDCTHLYRILLK
>MsexOBP27	MKRAKRRQPRQETNMFKIDFQSLMLFLIFGAAFILVIAFQPLSKEEHIERYN

	KMSEDVEPFRKNLTECARQVKASMVDVEHFLKRIPQSTMQGKCFVACILK<br>RNNIITKNKVNVKNLLEANRAVYGDDSEVLARLKTAVKECSEVVEGVFEI<br>CEYASVFNDCMHMKMEHLLDRMTMERRMEALGQMTSNPDEWSDDEDE<br>MLKLVKDEL
>MsexOBP29	MMGVDAIHDPQTKIDKDTIITRNLKLEKKGQAKTSVQNRKKDIEREPDWS<br>YQPIPPEVSTHVEQFKKNMTECLKEVQANDKRPVKRLSPKTESPIHGECLI<br>ACVLKRNGVIDNGKINKNNLLTLVSKFYAKDTKLMKKLDKNLEHCIEISTR<br>NRDECVLASQLNACTNDLMASNKHKIIVNY
>MsexOBP7	MYFKILVLCGILAIKNGICDERRAVSFVPEVADHITKTIKYCMVKHDNDPKI<br>IELVRQGSYGVDEPFKKFIHCAYYKSGYANEDGHVLVNKVIKAFPKDANIE<br>EVTKKCSTIKGEDAEDTTYQFFKCFELNAPIRLALE
>MsexOBP16	METFQFAVFCVAFYTFVVSSPIKEFYPKRDVATLKKYQMECVEDTKVDPDL<br>VIRFKAGDWRSEQPSLKNWVLCILNKMGLMTMDGVYRLDEAMARVGTK<br>DKDMAEKLIDQCLSTTALPAPDIAWKYVHCLHVNDPLGNYSSISILTP
>MsexOBP17	MQSCLFLTLLVAVVGINADTVLLTDIHKEKSNANIAACIKESGVKPEILAEA<br>KKGNYSEDEAMKKFLLCFFNKSGIMNADGKLNLDVALANLPPGVDKNEA<br>TKALEECQHKNGKDAPDTAFTIFKCYRAATKTQVLF
>MsexOBP18	MYQYNGYRSSSQSPRRYKRERRVDNTGQRSQYNPNTQRNSGYEDTYRNE<br>EKNSSENNTNTDNKACALQCFLENLQMTAQDGMPDKYLVTHAITKNVKN<br>DDLQDFLQESIDECFQILENEDSDDKCEFSKNLLLCLSEKGKANCDDWKD<br>DMHF
>MsexOBP21	MIKQMCAFLFALSVICSVNLAASKSIYVFPPEKAEMILENAIKCITESGLQT<br>FVGQEIKQGKYTDDERTLGALVCANEKIGYSNESGRLNIDKIMIDLFPLKP<br>EIRSDLEACNKDYGLDPVGTFKSFLICFRKRVPFRVVL
>MsexOBP22	MSWLHAVVALALAASAAPATTSCKNCIALGKEEKAMFRAHSDACLPQSG<br>VEPKVVESMLNGQLVESAALRRHVYCVLMKCKLVSKEGKLMKNAMLGK<br>MAMRSDGKNATKVLEGCADQTGDTPEDLAWNLFRCGYDKKTMLFDYM<br>PTSGASSGDIDNISK
>MsexOBP30	MYINMDFRVKLVTRCLTLKRHSYCKMKITASLVFVLLNVVSIYGESKEIYF<br>FPKEAAVTFLEATLKCIADSNYDSSVIDQIMQGKYIEDDKTVNALICACVAT<br>GFGYPDGKVNVEKIMKESVPTRQDLRPFIEDCNRESGKTPAQTFRGIVKCY<br>REKLPVQLRFSN
>MsexOBP31	MQEVEYADERRNVTDILKETDVPNSTSFNDEGFTTRNLEDAALDSTKIQII<br>RKYNDTDTISRRKKRSEPLFDKPDTNQCLSQCVFANLQVVDSRGIPRETEL<br>WNLVQSAVTSQQSRAALRDQIRACFQELQSEAEDNGCSYSNKLERCLMLR<br>FADRKMDEGNANEKKADQ
>MsexOBP32	MLCMHHCKLTIFVTTINMQSYLFLTLVLAVVGINADIVLLTDVQKEQSNAN<br>IAACIKESGVKPEILAEAKKGNYSEDEAMKEFLLCFFNKTGIMNADGKLN<br>LDVALANLPPGVDKNEATKALEECQHKNGKDAADTAFTIFKCYSAATKTQ<br>VLF*
>MsexOBP33	MKAAQNLFGIILFSVFAAVIAASTKKIFHLSPESGEKLVEGIIKCIAKLNFDPS<br>LINLIKEGKYLEDERLIKAIICMNVDSGVGTADGRLNVDAVMERIFSNNAEI<br>RKGLICCEKEYDGTPVGNLRGTLTCLKETLPFKIRM
>MsexOBP35	MRTAIFLLLVSVISCYAAEEKDTYYYPKAPSEIFLNAAKKCVEDLSYNSSIM

	DQIMQGKYIEEDKTLNVLICAAVNTGYGNADGKLNVEKVVKELYPERQD VWPIIEKCNLEQVTSTPLETFKGIVVCLKNNLPFKIRFPM
>MsexOBP36	MKVTILVILIIGVSCDAAFTKPIYRFPPKQSEMYVEAVVKCVAKLGYDLAIL DQIRQGKYTDDDKSVEALVCANNDIGYGLPNGQLDADKTIQDLFPTKPEI KSVFDKCDKDYGVDPAGNFKAFLLCFKDEIPFKVII
