# Supplementary material for: Pheromone-Binding Protein 1 Performs a Dual Function for Intra- and Intersexual Signaling in a Moth
Source: Int J Mol Sci. 2024 Dec 6;25(23):13125. doi: 10.3390/ijms252313125 (PMC11642448; doi:10.3390/ijms252313125)
Supplement: Supplementary file 1 [file ijms-25-13125-s001.zip › Table S2.pdf]

Table S2 Amino acid sequences of OBPs of *A. aeneociliella* and other insects used for motif pattern discovery

Gene name	Sequence
>AaenPBP1	MWEKMGIKMFVVVLLGMSVSVDSSQTVVKSMTKYFFKAYEVCTKEY<br>NIKEGTLGQIFNFWREDFTTNSRDIGCTIYCLSTKLDLLDPEGKLHHGN<br>AAEFAMQHGSDEATAKKLVEILHTCEQTTPPNDDKCMKALDVAFCFKK<br>ELHKLDWAPDSEVLFEEIIAELG
>AaenPBP2	MCMSDKLDLVDPEGKMHHGNALEFAKKHGADDAMATQLLDLLHKC<br>EDANPAGDDKDDCARVLEIAKCFKAEIHKLNWAPSMDLIMAEVLADV
>AaenPBP3	MMNSMLGAHLSSCMCPWKHLATSRILSQWSSFASFCFSHPCSSDIICFA<br>VSSSRERKTSF
>AaenGOBP1	MKDITLGFGAALEHCREESGLTEEKMEEFFHFWREDFQFEHRELGCAIT<br>CMSRYFNLLTDADRIHHLNTEAFIKSFPNGEKLALQLVEVIHQCEKKFD<br>SEQDTCWRVLHIAECFKDTCRERGIAPAMEMLLAEFIMEAER
>AaenGOBP2	MACSWMCLGLVVMAAVGSVRGTAEVMSHVTAHFGKALEECREESGL<br>SSDIMEEFKHFWSDDFEVVHRELGCALICMSNKLSLLHGDTRVHQINM<br>HEYVKNFPNGEVLSEKMVTLLKNCEKQYDDITDDCVRTVKMAACFKE<br>DAKKEGIAPEIAMIESVIEIYE
>AaenOBP1	MCLLSIISVSSRHGNKRTCSKMFKEAVLIGLWLVVACRSDTPVAPPAPPQ<br>VYCGEVPSQMYNCVKLPKIIGENVTSKCGGSSSPCERVTCTFRESGFLV<br>DGKVDKKKVSEHYDRFEAEHPEWTVAVRHVQAACLERDLPSQGLYLN<br>CPAYDVMWCAFTSFIRSAQPSQWASTEKCAYPRQYSAACPVCPSECFAP<br>AVPVGACNACLALPRTP
>AaenOBP2	MVRQISALLCCLCVFGISLSDSAISAESEKRCRNPPTAPQKIERVITLCQD<br>EIKLSILREALDVIKEEHTMPAQKRRNKREVPFTHDEKRIAGCLLQCVY<br>RKVKAVDGYGFPTLEGLVGLYSDGVNERGYFMAVLEASRECLMRHHD<br>HFSRTVPMDNGRNCDVSFDIFECISDRIGDYCGNSGL
>AaenOBP3	MYKLCVIIIAVVCARYVDAVSEELQTLIQSKLISAGLECIKDHPLSLSDIR<br>AFKDKRMPNNEDAKCFAACLFKNLGIMDDMGKLNPSTASESAKQVFR<br>NNEEHRKKADQIVRVCSKVNEQMTSDGNRGCERAKLAFECLTKNAAR<br>FGFDFDFS
>AaenOBP4	MARSTILLCSLYFFALTPYLTKAMTPDQRAIIKAHFEQLGMECIKDYPIT<br>EDDISDLRGKKIPSGPGAPCFLACMMRKIGVMDDAGMLQKEDVLELA<br>KKVFEDEEELKIISDYLHSCHQINDAAVTDGAKGCERAMLALKCMTEN<br>APKFGIEM
>AaenOBP5	MAKFSILCLAVLAAALNVKALTQDELSEIKAAMMVHFTECNKEFGVSE<br>ETLKEAKEEKNLDKIDPCLIGCVFKRSGAVDAKGLFDTEKVLEMSKTY<br>LKTDEDQSKFAEIVKDCAKANDEKVPDGEDGCERSKLVAICFAKHKDE<br>LVSSRR
>AaenOBP6	MFKILCLVFAVYYLQEVKSMTEMEAKLEFTKFVMKCMKEHPVEPTDL<br>ASLQQYMLPKNPEVKCLLACAYKLDGIMTQDGLYNKEHAYKIAEKMK<br>NDDEKRLENGKKMADICAKEVNEAQVSDGEKGCDRAGHLFKCTIDN

APKFGFKLV
>AaenOBP7 MSKVGVRFVVVACILEFALALSTEQIGEIQSKFITVGEACIKDHPLSEEDI
QAFKYRKFPEGENAPCFSACVLKNIGFFDDKGILSRETALEHAKKIFQA
GNEIKTVEEFIETCSKVNDEEVTDGEKGCERAKALFSCFAENLEKFDF
>AaenOBP8 MIFKVFSVFLFVLATSECRSVSEIKQWIMKKALSCSSEVAVTSEEQQML
LAHKMPNTKGAKCFTACVFKKVGWMNDKGMYDDAKAHEFATEEYA
NDATKLEASKKIFTICKTVNDMTDAPDGCERAYGLARCLLENAPKVGF
NLDVL
>AaenOBP9 MAGLVLKIFLVSIIVNCAKAESLEDLKKQYTSTLVECAQKYQLTPGDIL
QLQDKKMPDTESAKCMFACAYKASGMMDDHGMLSVEGLKRINEKYL
SDDTEKMENAFKFVDACKSVNDQKVSDGDKGCERAALIFQCSVEQAA
VFNLE
>AaenOBP10 MYSLFFYFVIIISASYGDLLTQERNRGATLKPISACCDIPELGDAKPLTEC
SNPKLLGPCNDVQCVFEKAGFLVDRNTLNKDVYKRHLRKWAEEHQG
WADAVERAITDCVDKDLRQYLDYPCRAYDVFTCTGIAMLKKCPKDAW
KC
>AaenOBP11 MYYSWFVLFVYLSVLLHQVVPENDHENPFSSIVKKTLIATAHSCMDKV
DATQADLEYLRSDPPYPEKASCIIKCLLEKVGVVKRNKYSKTGFMDAV
TPLVLKNKKKMAHMNTVSENCEKEINHHEVTPCQLGNEVITCIYKYA
>AaenOBP12 MCSMGYRRVVMVLAMAAAVACMDEEMAELAKMLHDNCGEETGAD
LSLVDKVNAGADLMPDPKLKCYIKCIMETAGMMNEGEVDVEAVLALL
PDDFRAKNEKSLRGCGTQRGADHCDTAFLTQACWQKANKADYFLI
>AaenOBP13 MYGFLFFVLIIVSVLALDLNEYEQKCGKKLMMTKECCKSVPELPLDIK
NNMKECNQLPGHTCEREICMAIKMGTGTAEGKLIMDKAIENIENIFKDS
KEIMDAIKSECIEKDLKMYGTPDTCEIKNWKLCINVQLIKTCAEWLED
DNCKALKENSQECAQLFQ
>AaenOBP14 MTWILLLGLLGVIGGVYPATTGCKNCITLGKEEKAMFRAHSDACLPQS
QVDPKLVEGMLTGELADDEALKRHVYCVLLKCKVISKDGKLQKTAVL
GKMATKADGKNATKVLEGCADQTGDTPEDIAWNLFRCGYDKKAVLFE
YMPTNISSSDVEIL
>AaenOBP15 MKTFIVFAVCLVVALALTDEQKEKLKKHKTECLAETKVDEQLVSKLKA
GDYKSDNEALKKYALCMLIKSELMTKEGKFKKDVALAKVANPADKPQ
VEKLIDTCLANKGNTPHQTAWNYVKCYHEKDPKHAIFL
>BmorGOBP1 MWKLVVVLTVNLLQGALTDVYVMKDVTLGFGQALEQCREESQLTEE
KMEEFFHFWNDDFKFEHRELGCAIQCMSRHFNLLTDSSRMHHENTDK
FIKSFPNGEILSQKMIDMIHTCEKKFDSEPDHCWRILRVAECFKDACNKS
GLAPSMELILAEFIMESEADK
>BmorGOBP2 MFSFLILVFVASVADSVIGTAEVMSHVTAHFGKTLEECREESGLSVDILD
EFKHFWSDDFDVVHRELGCAIICMSNKFSLMDDDVRMHHVNMDEYIK
GFPNGQVLAEKMVKLIHNCEKQFDTETDDCTRVVKVAACFKKDSRKE
GIAPEVAMIEAVIEKY
>BmorPBP1 MSIQGQIALALMVYMAVGSVDASQEVMKNLSLNFGKALDECKKEMT
LTDAINEDFYNFWKEGYEIKNRETGCAIMCLSTKLNMLDPEGNLHHGN
AMEFAKKHGADETMAQQLIDIVHGCEKSTPANDDKCIWTLGVATCFK

AEIHKLNWAPSMDVAVGEILAEV

>BmorPBP2 MKLQVVLVVLTVEMVCGSRDVMTNLSIQFAKPLEACKKEMGLTETVL
KDFYNFWIEDYEFTDRNTGCAILCMSKKLELMDGDYNLHHGKAHEFA
RKHGADETMAKQLVDLIHGCSQSVATMPDECERTLKVAKCFIAEIHKL
KWAPDVELLMAEVLNEVSWKS

>BmorOBP5 MKQRLRVLLLRFCILQTVLSESGVDVVKNLSLSFARFFLECDEERHFQP
EVRLKVMTFWYSESSTWDRDVGCAFLCIFKKMEIDNPQDPSYRTHLEL
LSFANSEDNKIANQMVEIFYACGENTETDPCLWALEQVKCYKNRINQL
GLTPTF

>BmorPBP3 MARYNIVVAVLVLGVVGARGSSEAMRHIATGFIRVLDECKQELGLTDHI
LTDMYHFWKLDYSMMTRETGCAIICMSKKLDLIDGDGKLHHGNAQA
YALKHGAATEVAAKLVEVIHGCEKLHESIDDQCSRVLEVAKCFRTGVH
ELHWAPKLDVIVGEVMTEI

>BmorOBP7 AVTEEELKIEFTKLVMKCTKDHPVDMSELMQLQQLIAPKKTESKCLLA
CAYKLNGVMTSQGLYNLEHAYKIAEMSKNGDEKRLENGKKVADICVK
VNDVEVSDGEKGCERAALIFKCTLENAPKVFKFGSSEYNCQ

>BmorOBP8 MLRVVVICVCFLVIAPYGINASSLDDLKMVYKNVIKECVGDYPITAADL
KLIKARQIPNDDIKCVFACAYKKTGMMTEEGMLSVEGIKDMSQKYLSD
NPEQLRKSKEFAEACSSVNDQQVSDGTKGCERAALIFKCSTEKITNFGF
EL

>BmorOBP9 MLRVVVICVCFLVVAPYGINAVSYEQKIKIRDQLDRAGFECFKDHKITE
DDIKNLRANKPATGENVPCFIACVMKKTGVMNDQGVIRKGPVLELAK
KVLADDKDIKKLQDYIHSCSHVNSETVHDKGKGCEFAMQAYTCMSAN
ASKFGFNI

>BmorOBP10 MLRVVVICVCFLVIAPYGINAVSDEQKIKIREQIDKSGFECFKDHKITED
DIKNLRARKPATGENVPCFIACVMKKTGVMNDQGVIHTEPVLQLAKK
VLTDDKDIKKLQDYIHSCSHVNSKTVHDKGQGCEFAIQTYTCMSANAS
KFGFDV

>BmorOBP11 MSANSFVVLAFCALAVGVNALTEEQKAEITKSSLPLIAECSKEFSVNQG
DIDAAKKLGDPSGLNSCFVGCFMKKAGIINASGLFDVAATIEKSKKYLT
SEEDLKAFEKLTETCAPENDKPVSDSDKGCERAKLLLDCFVANKGSFS
VFSL

>BmorOBP12 MTSFMVFFVLSVLTLKYSDALTDEQKNKIQSKFIEIGAECIVEHPISIDDI
NSFKNKKFPSGVNAGCFVACIFNKIGLFDDKGNLSHNSALEKAKGIFNA
DEEVKNLEEFLNRCAKVNGEAVGDGVKGCERAKLAYNCLIENSLEFGF
NIDF

>BmorOBP13 MLKIHVLLCFGMAILYFGSAKAVTPEESKAFEAFAKPVIEQCQKDFGM
DKESFAQKNLDEIDECLIACVVEKFGITNDEKIDGDALKALVTKFVGNE
EERNKINKIVEECTEDANKSGDGTCNTSTILFLCLLKNGKDLWGF

>BmorOBP14 MERKDFYLLIVVVALTSGVSSMSRQQLKNSGKMLKKQCMGKNDVTEE
EIGDIEKGKFIEQKNVMCYIACIYQMTQIIKNNKISYEASIKQIDLMYPPE
LKESAKASAGRCKDVSKKYKDICEASYWTAKCMYEDNPKDFIFA

>BmorOBP15 MFLKNIFIECVLLYFVMLNTSFVNTMTKQQIKNSGKILKKACISKNDVT
EDQISDIDKGKFIEDKNVMCYIACVYSMSQVVKNNKFVHDAMVKQVD

MMFPTEMRDAVKASIANCRGVAKNYKDICEASFWTAKCMYEFDPANF
VFA

>BmorOBP16 MRISFLFLISVTIITFDSVFAMTRAQVKKTMTIMKNQCMPKNGVTEDQV
GKIEEGIFLENHNVMCYIACVYKTIQVVKNDRLDKDLISKQIDVLYPQE
IRESTKKAVGDCINLQEKYDDWCEGIFRSTKCLYEKDPANFIFP

>BmorOBP17 MTRQQLKNSGKIMKKTCMPKNDVTEEEIGQIEQGKFLEQRNVMCYIA
CIYTVTQVVKNNKLSYDAVIKQVDVMFPAEMRPAVKAAAENCKDISKT
FKDICEASYWTAKCMYDFDPKNFVFP

>BmorOBP18 MILIVIAKFLILISLCETMTMKQIKNTGKMMRKSCQPKNNVDDEKINPI
NDGVFIEENEVKCYIACIMKMANTMKNGKLNFEAAMKQADLLLPDE
MKEPTKEAIVACRKVADSYKDVCDASFHVTKCIYNHNPSVFFFP

>BmorOBP19 MTSAKTDVEIKAWFLGQAVECSKDHPVTTEELRMHKHELPDSKNAKC
LMKCVFRKCNWLDSKGMYDINAAYASSTKDFSDDKTKQENANKLFD
TCKSVNEENVGDGEEGCDRSLLLAKCLTKAAPQVSIYYS

>BmorOBP20 MAVHIFLILASYMALAAHGQLDDEIAELAAMVRENCADESSVDLNLVE
KVNAGTDLATITDGKLKCYIKCTMETAGMMSDGVVDVEAVLSLLPDS
LKTKNEASLKKCDTQKGSDDCDTAYLTQICWQAANKADYFLI

>BmorOBP21 MITASLHVIFALLAFVYGGKDKPVLSEEIKEIIQTVHDECVGKTGVSEED
ITNCESGIFKEDVKLKCYMFCLLEEAGLVNDDGTVDYEMFTSLIPEEYF
DRATKMIFSCKELDTPDKDKCERAFEVHKCSYEKDPDFYFLF

>BmorOBP32 MYSHKYLNDFTNIPEILIILLSSVALMSYGYNTKLFSHSLGSEPSLSILYA
RDKKSDKVTNECLMEMYPKNLYKYPLRIDRNDIPCIIHCVLKKFGIISN
DGFINIKNYYRRVQAIHRYDPRILISDVGETCAQNINGMNLDHDVCKK
AKVFNDCTQLYAISYREPEDW

>BmorOBP33 MYAHDKLSDMIADQCLNEMYPRSKRLEIEESDEPCIIFCVLKKFGIMSP
TGVINLEAYRKRVQLPEQLAQRNSINDFGSACLESAEATQHKQDVCKK
AKVFNECTHLYKILLK

>BmorOBP34 MEKMILLNVFAVVLPCVLASRTRGSSGTLVDFTDPKVQGHLDALVRMA
QSCVIKVRATPKDVRAYFTNSSPVSRSGQCFATCMLEQSDIINHGKVNR
DLLVHLAGLVNGKNSRVVRKLNSVSRLCLDSISGMTDRCQLASTYNDC
LNENMIEFAFPLDIAEEAVRKMPFHLIQPK

>BmorOBP35 GMSTHVLDFKRNMTECLKEVQNNDKRPIKRLSPKQESPIHGECLIACV
LKKNGVIQNGKVNKDNLMALVSKFHAKETKLMKKLEKNLDRCINISV
KNHDECSLASQLNDCTNDIMASSKQKILFNY

>BmorOBP36 MAVSEISRILTFLTIVSFIYIVYSFKPLTKDEHIERYNKMNEDIEPFRKNLT
ECARQVKASMADVEKFLKRIPQSNMEGKCFVACILKRNSLIKNNKLSQ
ENLLEVNRAVYGDDSEVMSRLKTAILECSKIVEDIFEICEYASVFNDCM
HMKMEHILDKITMERRMEALGQMSSNPDEWSEEEDEMLKLVKDEL

>BmorOBP37 MFYPFRFTLLFYGLFVIYLVRAEPEKENHFTLALKKTLFSTARSCMSHV
NANETDLEYLRKDPPFPDKAACIIKCLLEKIGVVKNNKYSKMGFLTAVS
PLVFTNKKKLDHYKSVSENCEKEINHDQTTECELGNEVVSCIFKYAPEL
HFKT

>BmorOBP38 MANLVLLLTFVLMTLSMARLKSTEAPKSKTALFNDQDNMGYEELDME
EIMSACNESFRIEYAYLESLNDSGSFPDETDKTPKCYIRCVLEKTEILSEN

GVLNPATAALVFAGERNGKPMSDLEEMAVACADRHEKCKCEKAYNFV
KCLMYMEIDKYEKKN

>BmorOBP39 MVRKISALLCCFCVLGISMCDSAISTDNEQRCKNPPTAPQKIERVITLCQ
DEIKLSILREALDVIKEEHTMPAERKRNKREVPFTHDEKRIAGCLLQCV
YRKVKAVDGFGFPTLEGLVGLYSDGVNERGYFMAVLEASRECLMKNH
DKFSRTTPMDNGRNCDVSFDIFECISDRIGEYCGTSGL

>BmorOBP22 MLKVFVVVVCTLGASQLCAALYTQKVAVSFPKDKTTIVVEAMKSCIAK
TGANPNVIEVISSGKVSEDEKFKEFFYCACNDIGVVNPDGHIKVKECIE
LFPKETQPLVEPVIKNCDKEGVNKYDTLFKYLKCFQETSPVRVTLA

>BmorOBP23 MTSKVLLSCVVLAVLATTVLAEDSRKLVSFAPEVAKKLKVLIQECLNEN
GLGEDAIEVIRAGEYREDEPFQNLVYCAYKKFGALDENNRIISQVAAAS
FPKDIDVVTVIESCGKEDGNTPVEQVFKYFKCFQKNSPVRMQLY

>BmorOBP25 MKSVVLICLAFAVFNCGADNVHLNEDEREKANWYTAECGVETGVSTE
VINAAKIGKYSKDKAFKKFVLCFFKKSAILNSDGTLNMVVALAKLPSG
VNKSEAQSVLEQCKNKTGQDAADKAFAILQCFHKGTKTHILF

>BmorOBP26 MKSVVLICLAFAVFNCGADNVHLAETQKEKAKQYTSECVRESGVSTE
AINAAKIGKYSKDKAFKNFVLCFFNKSAIFNSDGTLNMDVALAKLPPG
VNKSEAQSVLKQCKNKTGQGAADKAFEIFRCYYKGTKTHILF

>BmorOBP27 MKSVVLICLAFAVFNCGADNVHLTETQKEKAKQYTSECVKESGVSTEV
INAAKTGQYSEDKAFKKFVLCFFNKSAILNSDGTLNMDVALAKLPPGV
NKSEAQSVLEQCKDKTGQDAADKAFEIFQCYYKGTKTHILF

>BmorOBP28 MLKVFIVTFFAFQLSAIARLQANGCVAVPFPKDKTIIIVEAMKSCIAKTG
ANPNFIDVIRSGKVSEDEKFKEFYYCTCNDTGFVNPDGHIKVKECIELF
PKETQPLVEPVIKNCDKEEGVNKYDTLFKFLKCFQETSPVRVALA

>BmorOBP29 MTGPAAAAVLLALLAAAGQATTGCKNCVILGKEERAMFRSHSDACLA
QSRVEPRLLESMMNGELIDDAALRKHVYCVLLSCKMIGKDGKLLKAAI
LGKLAARPAGRDVTKVLEACAEQPGASPEDVAWNIFRCGYNRKAVLF
DYMPAGGASSGNTENHP

>BmorOBP30 MRSFVILLNYGLLCCGQFMAEDYYYDIVTRDPDDLMREKENEVRALR
AFQADCAEDVQVKPDLVVNLKSGDWQTEDVSLKKWALCVLMKLGL
MTAQGVFKMNEAMSKIPDMNDKIIAEKLIDDCLSLQATTPHDAAWNYI
KCHHQKDPEGNFSSLNIF

>BmorOBP31 MKTFIVFVVCVVLAQALTDEQKENLKKHRADCLSETKADEQLVNKLK
TGDFKTENEPLKKYALCMLIKSQLMTKDGKFKKDVALAKVPNAEDKL
KVEKLIDACLANKGNSPHQTAWNYVKCYHEKDPKHALFL

>BmorOBP40 MSEFIQPSWRTQCNFRLNWDNRNRLSIDISHGAATTQTPVPTTKPKALR
DFMVVPQSCDKTTCVFKKLNIVSDKGVVDVKSFIKLLDKFTNSYPVW
NSAKARVITTCLRKSLIAYDGGCELNNILACTFDVLSENCPLNGNNQTC

>BmorOBP41 MLTILFLLPIVVGVLSGNIPEQPRVYCGELPNTIYSCLGNPKIIQPEVSEK
CNKPISECDKTRCIFKESGWAKNNVIDKKKVSDYFEQFAKDNPDWSAA
VQNFKTTCLSDSLKPQGVDTNCPAYDIIHCALISFIKFASPSQWSTSEQC
VYPRQYAGACPVCPERCFAPSVPNGSCNACLALLRTP

>BmorOBP42 MMGYACVFVILAVLQAISAEDPPGLPPFLKDAPEKCKSPPRVKNPNECC
ISEPFFKEADFIECGIEKPGSERGPPDCSKQNCLLKKYNLLKNDETPDIE

AIKSLLDKYIEKNPSFKSSVEKAKECLREDLPGPPQICLANRMTLCIGTV
LLMECPDEKWNTTDDCKAFKDHMTECQKYFPK

>BmorOBP43 MKVCVLFAIFTVAQAAKATLKPISACCNIPELGNPEPLAECSNPKLPGPC
KDIQCVFEKSGFLTENKTLIKEAYKTHLRQWAKEHEGWSVAVEKAISD
CVDKDLRQYLEFPCSAYDVFTCTGIAMLKKCPNEHWTC

>BmorOBP44 MSRLVLFFTILVVLQEFIINLYFNFITEIDSCCVKKYPKLFDSEFITECYNT
QRKANDKCERDMCVARKLNLLTEEDSINKDALLRFVEEGFKTEIDLVN
AIKKKCFEEDISNIGKPEMCEVAKYKICITSRMAEDCPKWDSKGICSSA
QQKVENFMKMLS

>SlitGOBP1 MLLLLALPLLAAVLPLRADVNVMKDVTLGFGQALDKCRQESQLTEEK
MEEFFHFWRDDFKFEHRELGCAIQCMSRHFNLLTDSSRMHHENTEQFI
QSFPNGEVLARQMVELIHACEKQHDHEDDHCWRILHVAECFKQACVQ
RGIAPSMEMMITEFIMEAEAR*

>SlitGOBP2 MATVTSSVMGTAEVMSHVTAHFGKALEECREESGLSAEVLEEFQHFW
RDDFEVVHRELGCAIICMSNKFSLLQDDSRMHHVNMHDYVKSFPNGH
VLSEKLVGLIHNCEKQFDSMTDDCERVVKVAACFKVDAKAAGIAPEVA
MIEAVMEKY

>SlitPBP1 MANARWRFVFVVYALYLTSAVLGSQDLMAKMTKGFTRVVDDCKTEL
NVGDHIMQDMYNYWREDYQLINRDMGCMLLCMAKKLDLMDDQTM
HHGKTEDFAKSHGADDDVAKKLVSVIHECEQQHTGIADDCMRVLEVA
KCFRTKIHELKWAPSMEVIMEEVMTAV

>SlitPBP2 MSLRVALVVAASLLVVVQASQDVMKNLAINFAKPLDDCKKEMDLPDS
VTTDFYNFWKEGYELTNRQTGCAILCLSSKLEILDQELNLHHGRAQEF
AMKHGADEAMAKQIVDMIHTCAQSTPDVAADPCMKTLNVAKCFKLK
VHELNWAPSVELIVGEVLAEV

>SlitPBP3 MGSRNVFVALVVLTVAMRETEPSKDPMKYIASGFVKVLEECKHELNM
NDHLIADLFHYWKLEYTLLNRDTGCAIICMGKKLDLLDANGRMHHGN
AQEFAKKHGAGDEVASQIVQIIHECEKKHERDDDECLRVLEVAKCFRT
GIHELNWQPNVEVIVSEVLTEI

>SlitOBP5 MTKVLFAIVLTMVTFAVVLSASTKEAMTTTMSDQVNSIDVDVLAVMD
MCNDSYRIDPTYLQALNESGSFIDETDKTPKCFIRCVFENVGIVSEDGK
QFNPARAAVIFAGERNGKPMEDIADMTALCATDRQETCPCDRSYKFLR
CLMSMEIERYEKS

>SlitOBP6 ESKFGEIVKRTVIATAHTCMDHVNATAKDLEHLRDEPPYPETSACIVKC
LLEKVKYMRKQTN

>SlitOBP7 MFTEALPLFVILVAVTHGGKNKPVFSDEIKEIIQTVHDECVAKTGVAEED
ITNCENGIFKEDAKLKCYMFCLLEEASLVDDDDTVDYDMLVSLIPDEY
YERTTKMIFACKHLDTPDKDRCQRAFEVHKCSYEKDPDLYFLF

>SlitOBP11 MSKFTCLVLCVVAVSLNGVHATAEEKAAFIEAVKPYVQECSKEHGVTP
EDIKSAKAAGNADGINSCFLSCVYKKAEVINEKGEYDVDKALEKLKKF
VSNEDDYAKFANIGKKCASVNEKSVSDGEAGCERAALLTSCFLEHKSEI
SA

>SlitOBP12 MSVVRCSSFLVALFCFVSVNAMSGDEEAGIKEALRPFVQECADEFGITE
EQFEEAKKKASAADIDPCFMSCFLKKAEFFDSQGKFDVDSTMAFAKEH

LTSEPAMKFVEAVGDECVKINDEDVSDGDKGCDRAKLLFECIAETKKK
ME

>SlitOBP14 MDQKRICLFVIAMFLASGSDAMSRQQLKNSGKMLKKNCMNKIGVTD
DQVGSIDKGKFIEDRKVMCYIACIYELTNVIKNNKLNYEASIKQIDLMY
PPDVKESAKAAVEKCKDVQKKYKDICEASFYAAKCMYEYKPEDFIFA

>SlitOBP15 MFNNCFVYSMTREQIKNSGKLIKKTCSAKNDLTEDEVKDVDKGKFIEK
KDFMCYIACVYKMGQTVKGSTINHDMMLRQVDMMFPNDMKAPVKA
AIEHCRPVAKNYKDLCEASYWTAKCIYDFDPANFMFP

>SlitOBP17 MRTFRLLCCILSIFFIFDQSYGMTRQQLKNSGKLMKKSCMPKNDVTED
EVGDIEKGKFIETRNVMCYIACVYTMSQVVKNNKLSYEAVIKQVDVM
FPAEMRDAVKAAATHCKETTKKYKDLCESSYWTAKCMYDYDAQNFV
FP

>SlitOBP18 MILXYTQKLTNMLLTKIVKFFILVATCEAMTMKQIKNTGKMMRKTCQP
KNNVEDEKIDPLSDGVFIDEKEVKCYMACIMKMANTIKNGKLNYDAA
MKQADLLFPDDIKEPAKEAITACRKVADAHKDICDASFHVTKCIYNHN
PSIFYFP

>SlitOBP20 MWVQALVLTLATLVTLVAAAVEMDEDMAELARMVRDNCAGETGVDV
ALVEQVNAGAELMPDDKLKCYIKCTMETAGMMADGEVDIEAVLALLP
PSLAEHNAPALRACGTQRGADHCDTAFRTQQCWQNANKADYFLI

>SlitOBP21 IHYLCAARAVHKIKNITNCENGIFKEDAKLKCYMFCLLEEASLVDDDD
TVDYDMLVSLIPDEYYERTTKMIFACKHLDTPDKDRCQRAFEVHKCSY
EKDPDLYFLILRREQLASRDDCVAISGIN

>SlitOBP3 MKSFVVICIVFVVGVCATEKGNKIASECIKESGVKSDVLAEAKKGNLG
DDPAFKEFTYCFFKKVGIVGEDGKLNRDVAIAKLPSGVDKAEAEKLLD
SCKSKTGKDAVETVYEIFKCYQHGTKSHIMFAS

>SlitOBP4 MKTLLVFAACILVAQALTDEQKEKLKKHRTECLTETKVDEQLVNKLKG
GDYKMDNEALKKYALCMMMKSELMTKDGKFKKDVALAKVPNPADK
PTVEKLIDACLANKGNTPHQTAWNYVKCYHEKDPKHAIFL

>SlitOBP1 MFKLCVFLALGFVACHGASNSNPGTPNANPGTYCGVTPDNIYRCLNNP
RVVTPEVSTKCGSQFTECEKMTCIFRELKWSKRGAIDKAKVRAYFDQY
ETEHPEWAQAVQHVKAFCLASELRAQGVFLNCPAYDIMQCVLASFIKH
ASPSVWSTATDCAYPKAYAADCPVCPSDCYSPQIPYGSCNACYTQPRTV

>SlitOBP2 MVRKISGLLCCLCVFGISFSDSAISADSESRCRNPPTAPQKIERVITLCQD
EIKLSILREALDVIKEEHTMPAQRRRDKREVPFTHDEKRIAGCLLQCVY
RKVKAVDGYGFPTLEGLVGLYSDGVNERGYFMAVLEASRECLMKNHD
KFSRTVPMDNGRNCDISFDIFECISDRIGEYCGTSGL

>SlitOBP13 MITSSSLLVLTAVVQVLFAQQPVFESGPPEPWGPPQRPAHRRQFLPRIPK
RCWVPPQRINVYNCCPIPTLYPDEDMQSCGFEKTSGNTDQPQKPVFRPE
GTCKEGYCVMGKFDLLFANNSVDFVKFREYLDNWAESYPEFANAIRIA
KQECAQDGGPEVPPICEPDKLFLCLTSTIFWNCKLRDGDGCAALQEHM
NECKQYYTRQMEPTMKDIEVR

>SlitOBP16 MLAEELKDCFDGSGPKDPMKCEIDLCIAKKKGFATDDGKLDIKKFEEVI
TKDVGSDKDLLDEIKTNCINGDLNNYGPPEFCDFIKIKHCVTLHMMNH
CSEWSDDGNCKVVKELVGKCAKVI

>SlitOBP19 MYSKICILLFISYTCLVTADSVSFIKKCKWDDGKCAKESGQNVIQKFAA
GISEYNVGVSDPLHIEYVDASSPNMKLIVTDVVVTGLRNCEVKKIQRFE
DSSKLIVKLLCAAELNGKYDMKGQLFVIPIEGNGGLYSKVPKIQINAEV
DLNTKQGKDGKDHWIVKSWRHTFELKDKSTVKFENLFPDNEFLRTST
NELIAQNGNDVIIEIGANLIKAIVGKIVENIKKFFIAVPIEDLSL

>SlitOBP10 MKEGNRYSHERRITNDSGDQLMVINATDDDYSGYGSGNMGEKLLTSV
PRPATPSNNINKNNINRTKRNEPLLNRPDSDQCLSQCVFANLQVVDSKG
IPREAELWNKVQSSVTSQQSRSALHDQIQACFQELQSEAEDNGCSYSN
KLERCLMLRFSDRKVDGKGNAKKSSTEQTG

>HvirGOBP1 MPGVLRALLLLAAAAPLLADVNVMKDVTLGFGQALDKCREESQLTEE
KMEEFFHFWRDDFKFEHRELGCAIQCMSRHFNLLTDSSRMHHDNTEK
FIQSFPNGEVLARQMVELIHSCEKQFDHEEDHCWRISHLADCFKSSCVQ
RGIAPSMELMMTEFIMEAEAR

>HvirGOBP2 MTSKSCLLLVAMVTLTTSVMGTAEVMSHVTAHFGKALEECREESGLSA
EVLEEFQHFWREDFEVVHRELGCAIICMSNKFSLLQDDSRMHHVNMH
DYVKSFPNGHVLSEKLVELIHNCEKKYDTMTDDCDRVVKVAACFKVD
AKAAGIAPEVTMIEAVMEKY

>HvirPBP1 MMSVRLMLVVAVWLCLRVDASQDVMKNLSMNFAKPLEDCKKEMDLP
DSVTTDFYNFWKEGYEFTNRHTGCAILCLSSKLELLDQEMKLHHGKA
QEFAKKHGADDAMAKQLVDMIHGCSQSTPDATDDPCMKALNVAKCF
KAKIHELNWAPSMELVVGEVLAEV

>HvirPBP2 PKWVFARAFCLVLMMGSAMSSKELLTKMTGGFTKVVDHCKTELNVG
DHIMQDMYNFWREEYQLVNRDLGCMIMCMTAKLDLVGDDQKMHHG
KAEEFAKSHGADDALAKQLVGLIHGCETQHQAIEDHCSRTLEVAKCFR
TKIHELKWAPSMEVIMEEIMTAA

>HarmPBP1 MEFHRSTMMSVRLALVVAVCLFIRVDASQDVIKNLSMNFAKPLEDCKK
EMDLPDSVTTDFYNFWKEGYEFTNRQTGCAILCLSSKLELLDQELKLH
HGKAQEFAKKHGADDAMAKQLVDLIHGCAQSTPDVADDPCMKTLNV
AKCFKAKIHELNWAPSMELVVGEVLAEV

>HarmPBP2 MAASRWLFARAFCLVLMMGSAMSSKELLTKMTGGFTKVVDACKTEL
SVGDHIMQDMYNFWREEYQLVNRDLGCMIMCMTAKLDLIGDDQKM
HHGKAEEFAKSHGADDALAKQLVGLIHGCETQHQAIEDHCSRALEIAK
CFRTKIHELKWAPSMEVIMEEIMTAA

>HarmPBP3 MGSRHVFFALVVLAVSVRKAEPSKDAMQYITSGFVKVLEECKHELNLN
EQILADLFHFWKLEYSLLGRDTGCAIICMSKKLDLLDANGRMHHGNA
AEFAKKHGAGDEVASKIVTIIHECEKKHEQDGDECLRVLEVAKCFRTGI
HELNWQPKVEVIVSEVLTEI

>HarmGOBP1 MPGVLRALLVLAAAAPLLADINVMKDVTLGFGQALDKCREESQLTEE
KMEEFFHFWRDDFKFEHRELGCAIQCMSRHFNLLTDSSRMHHDNTEK
FIQSFPNGEVLARQMVELIHSCEKQFDHEDDHCWRILHVAECFKGSCV
QRGIAPSMELMMTEFIMEAEAR

>HarmGOBP2 MTSKSCLLLVAMATLTASVMGTAEVMSHVTAHFGKALEECREESGLSA
EVLEEFQHFWREDFEVVHRELGCAIICMSNKFSLLQDDSRMHHVNMH
DYVKSFPNGHVLSEKLVELIHNCEKKYDTMTDDCDRVVKVAACFKVD

AKAAGIAPEVAMIEAVMEKY

>HarmOBP1 MSKFTFFVLCVVAVSLSKVYASDEDKAKLHEALKPLVEECMKDHEVSL
DDLKAAKEAKSADGVKPCFLACVYKKAEVLNDKGEFDADHALEKLK
EFVSDEDVLAKVAEVGNTCKAVNDKAVSDGDAGCERAALLTACFLEH
KAEILV

>HarmOBP2 MMDRKRLCLLIIAMFLAQGSDAMSRQQLKNSGKMLKKNCMNKNQVT
EDQIGSIDKGKFVEDKKVMCYIACIFEMTNVVKNNKLNYDASIKQIDL
MYPPDLKESAKAAVEKCKDVQKKYKDICEASYWTAKCMYDFKPEDFI
FA

>HarmOBP3 MSKFTCFVLCVLAVSLGEVRSNALEKAAIRAAVYPLIVDCAKEHAVTL
EQLKAAKASHSAEGINPCFQSCVYKKTGIFNDNGEYDVANAKTKLQKF
VTDEDEYARIAEVGKTCASVNDKSVSDGAAGCERAALLTACFLEHRA
QIII

>HarmOBP4 MSKLTCVVFAAVAVVFSNVNADDETRASFRQVLGPLVMECRNEFGITE
DDLKKAQQERSPDALKPCFIACVFKKFGIITSAGKYDSDASISRIKDVV
KNDDLLAKLKSVGEKCNSVNDASVSDGDAGCERAALLAKCFIENKSE
LSI

>HarmOBP5 MSKFTCLVLCVVAASLSQAYASEEEKAAFREAIKPIVEECSKEHGVSHD
ELKSAKDNQNADNIKPCFLGCVYKKAEVFNSKGEYDVDKALEKLKKF
VSNDEAYAKFAEVGKKCASVNDKAVSDGDAGCERGALLTACFLEHKA
EVPL

>HarmOBP6 MSKFTCLLLCVVAVSLSKVHATEEEKEAIRAAVRPIMQECGKEHGVTL
DDLKAAKAAHSADGIKPCFQSCVYKKAGIFNDNGEYDIANAKTKLQK
FVTNDEEYARIAEVGKMCASVNDKPVTDGAAGCDRAALLTACFLEHR
AQIII

>HarmOBP7 MFRFGVLSFVVLLFCMESSYALSSEEELSIKEALHPFVVECAEEYGMTE
EMFEEAKKKGSAEDIDPCFMSCFLKKTGFFDDSGKFDAEKSISFAKEHI
TSESAIKFLEAGAGECVKINDEDVSDGENGCDRAKLLFDCLTELKKKM
SE

>HarmOBP7.2 MSRFGVLSFVVLVFCMENIYALSSEEELSIKEALHPFVVECAEEYGMTE
EMFEEAKKKGSAEDIDPCFMSCFLKKTGFFDDAGKFDAEKSISFAKEHI
TSETAIKFLEAGAGECVKINDEDVSDGDKGCDRAKLLFDCLTDLKKKM
SE

>HarmOBP8 MLLIEIVKFLTLVAMCEAMTMKQIRNTGKMMRKSCQPKNNVADEQIDP
IAEGVFNEDKEVKCYMACIMKMANTIKNGKLNYEAAIKQADLLLPDD
IKEPAKEAITACRKVADAYKDICDASFHITKCIYTQNPGIFYFP

>HarmOBP9 MCKFSVLFLYSAVMAVNIWSASCISEEDKAAIITAIAPLAQNCGSECGLD
NDDFEKYKEDGSDMDPCFKACLMTQMGVLDKEGKYDGKGLHKAME
EADYPGDKDDAQKFLDELDRCFDAKGDNSGSDEEAKMKRADVLFRC
MQDMKEK

>HarmOBP9.2 MCKCSVVFLYLAVMAINIWRASCLSEEDKAAIITAIAPLAQNCGSECGL
DNDDFEKYKEDGSDMDPCFKACLMTQMGVLDKEGKYDGKGLHKAM
EEADYPGDKDDAQKFLDELDRCFDAKGDNSGSDEEAKMKRADVLFQ
CMQDMKEN

>HarmOBP13 MFTGTLPLVVFLATFAYGGKEKPVFSDEIKEIIQTVHDECVAKTGVAEED
ITNCENGIFKEDPKLKCYMFCLMEEASLVDDDDAVDYDMLVSLIPEEY
VDRTTKMIFSCKHLDTPDKDKCQRAFEVHKCSYEKDPDLYFLF
>HarmOBP18 MKSFVVFCVLVAGAFAANVSLPPKQNEKANQIATECMKESGLKPEVLA
EAKKGHISDDEHLKKFTFCFFKKAGIVSEDGKLNTEVALAKLPPGVDK
AEAEKLLETCKGKTGKDVTDTVFEIFKCYHHGTKTHILLGF
>HarmOBP16 MFKLCVVLAFIVATCHGGTLERTSSTCGQIPRELTACLDLQPAVSPEIQE
KCRRANECERLTCVFREYNLLDGAEVNKERTAAFLDNFVKQYPSWEV
AIDVAKTSCLRSSGLKPQGVFLDCPAYDIIQCVFANLVKNALPSQWSSM
SQCNHAREFAAACPICPDACFAPLVPIGTCNACSAARRSS
>HarmOBP17 MRAWSVTLVALLGALGAARAVAMDEDMAELARMVRENCAAETGAD
VALVERVNAGADLMPDDKLKCYIKCTMETAGMMADGEVDIEAVLALL
PPELAEHNAPSLRACGTVRGADHCDTAFRTQQCWQNANKADYFLI
>HarmOBP18a MTRQQLKNSGKLMKKSCMPKNDVTEEEVGDIEKGKFIESRNVMCYVA
CIYTMTQVVKNNKLSYEAVIKQVDMMFPAEMRDAVKAAATSCKDITK
KSKDLCESAYWTAKCMYDYDAENFVFP
>HarmOBP22 MTREQIKNSGKLIKKTCMAKNDLSEDQVKDVDKGKFIEEKPFMCYIAC
VYKMGQTIKGNTVNHDMMIKQVEMMFPNEMKAPMKAAIEHCRPVV
KKYKDVCEVSYWTAKCIYEFDPPNFMFP
>MsexGOBP1 MGQNTRSLVLVVLVGLVGAVSADVQVMKDVTLGFGQALEQCREESQL
TEEKMEEFFHFWREDFKFEHRELGCALQCMSRHFNLLTDSSRMHHEN
TDKFIKSFPNGAVLSKTMVELIHNCELQHDAEEDHCWRILRVAECFKIS
CTKAGIAPSMEVMMAEFIMETENK
>MsexGOBP2 MVNRLILVVVVVFITDSVMGTAEVMSHVTAHFGKALEECREESGLPVE
VMDEFKHFWREDFEVVHRELGCAIICMSNKFELLQDDTRIHHVNMHD
YIKSFPNGQVLSEKMVQLIHNCEKQYDDIADDCDRVVKVAACFKKDA
KKEGIAPEVAMIEAVIEKY
>MsexPBP1 MKVAVVAIVVYLAVGNVDSSPDVMKNLCLNFGKALDECKAEMNLSDS
IKDDFANFWVEGYEVSNRDTGCAILCLSKKLDMIDPDGKLHHGNAME
FAKKHGADEAMAKQLLDIVHNCENSTPPNDDACLKTLDIAKCFKKEIH
KLNWAPNMDLVVGEVLAEV
>MsexPBP4 MKEAGVRFKILLFLIFPVVTGNFKGKQIMRSVAETFGRTVFECQNEVLM
KFGSGILNDIFRYWHEGQPLEDRDLGCIFRCILLKLELVNDNGRLIDAN
ADGFFQANGADESMTKHLIELYHSCYQTMRFPQDDCMLILEIGKCCRE
GVRNAHWTPGSK
>MsexPBP3 MAVIPIFTVLLMMTAVKEIAPSSDAMRHIANGFLKVLDQCKHELGLTDQ
IVVDLYQFWKLQYALLNRDTGCAIICMSKKLDLLDGTGRMHHGNTQE
FAVSHGATDEVASKVVVIIRDCEKQQEGEQDDCVRVLEVAKCFRTAIHE
LNWAPNMEVVVDELLTEI
>MsexPBP2 MVSTKWCFLLVTIAVLTMEVVSASQEVLKQMSVGFSKVLQTCKTELSV
GDHIIQDFYNYWREDYDLLNRDFGCMVICMAVKHDLINDQLTMHHGN
AHAFAKTHGADDDTAQQLVTILRECEAKHQSVEDVCNRALEMAKCFR
TKIHELKWAPAMEVVLEEIMTSV
>MsexOBP8 MCKPMYCVVFSIIYLSIVAAQKADNGNTKIANLQSNDQDSMDNVDVE

DIMNQCNETFRIEMAYLQALNESGSFPDETDRTPKCFLLCVLDNTGVM
MKDGDFDPERTAVLFAGERAGKVMDGIQDMAAACADRKEKCKCEKS
YNYLKCLMTMEIEKYANNN

>MsexOBP11 MWKISYFLFVFGVAANLKHAYAVTEEERLMLDSLMKPKVLACVEEFG
LKDFSIEDIRKDHEIDPCLLQCFLKKAEVFVDGMINLEKADETLREVIN
DEDEVEQIMEKGKECADEANGSDVSGDDEDCARVAIFHSCLREKNGLF
MATS

>MsexOBP12 MPKHDVTEDQVGGIEQGKFLENRNVMCYIACVYSMSQAMSKQQLKN
SGKMFKKQCMGKNKVTEDEIGEIDKGRFVEQQNVMCYIACIYQMSQV
VKNNKLNYEASLKQIDIMYPPELKDTAKGALEACKDIAKKNKDLCEAS
FKTAKCMYEYSPKDFLFP

>MsexOBP13 MLFLVVAKFFILLSLGEAMTMKQIRNTGKMMRKQCQPKNNVEDEKID
PLGKGVFINEKEVKCYMACIMKMANTIKNGKINYEAAMKQADLLLPE
DIKEPAKEALTSCRKVADSHKDVCDAAFYISKCIYEYNPDIFYF

>MsexOBP14 GITEKQRLHIREELIEIGTECIIKNPITIGDVRSFRNKQFPNGPNAGCFIAC
VFNKAGIMLGLLEINNFPTVQTQDALLRAYLIKQELFDDEGLVSQKTAT
EKATKVFDDETELKNYEQFIAVCDKVNEESVSDGQKGCERAKLAFQCL
IQNSKQVFIPYNLLLGSR

>MsexOBP15 MRRKLLNIKENPFTSVVRKALINTARSCMVHVNATQEDLEYLRKDPPF
PEKASCIVKCLLEKIGVVKNNKYSKSGFMMVVTPLVFANKKKLDHMK
TVSENCESEIVHKESSPCDAGNEVTTCIFKYAPELHLRG

>MsexOBP23 MYAHDKLSDMVAEQCLNEMYPKSKRIEIQESDEPCIIFCVLKKFGIMST
NGVINLEVFRKRVQNSHQHEQRNTMNDIGSNCLESAEATQHKQDVCK
KAKVFNDCTHLYRILLK

>MsexOBP27 MKRAKRRQPRQETNMFKIDFQSLMLFLIFGAAFILVIAFQPLSKEEHIER
YNKMSEDVEPFRKNLTECARQVKASMVDVEHFLKRIPQSTMQGKCFV
ACILKRNNIITKNKVNVKNLLEANRAVYGDDSEVLARLKTAVKECSEV
VEGVFEICEYASVFNDCMHMKMEHLLDRMTMERRMEALGQMTSNPD
EWSDDEDEMLKLVKDEL

>MsexOBP29 MMGVDAIHDPQTKIDKDTIITRNLKLEKKGQAKTSVQNRKKDIEREPD
WSYQPIPPEVSTHVEQFKKNMTECLKEVQANDKRPVKRLSPKTESPIH
GECLIACVLKRNGVIDNGKINKNNLLTLVSKFYAKDTKLMKKLDKNLE
HCIEISTRNRDECVLASQLNACTNDLMASNKHKIIVNY

>MsexOBP7 MYFKILVLCGILAIKNGICDERRAVSFVPEVADHITKTIKYCMVKHDND
PKIIELVRQGSYGVDEPFKKFIHCAYYKSGYANEDGHVLVNKVIKAFPK
DANIEEVTKKCSTIKGEDAEDTTYQFFKCFELNAPIRLALE

>MsexOBP16 METFQFAVFCVAFYTFVVSSPIKEFYPKRDVATLKKYQMECVEDTKVDP
DLVIRFKAGDWRSEQPSLKNWVLCILNKMGLMTMDGVYRLDEAMAR
VGTKDKDMAEKLIDQCLSTTALPAPDIAWKYVHCLHVNDPLGNYSSISI
LTP

>MsexOBP17 MQSCLFLTLLVAVVGINADTVLLTDIHKEKSNANIAACIKESGVKPEILA
EAKKGNYSEDEAMKKFLLCFFNKSGIMNADGKLNLDVALANLPPGVD
KNEATKALEECQHKNGKDAPDTAFTIFKCYRAATKTQVLF

>MsexOBP18 MYQYNGYRSSSQSPRRYKRERRVDNTGQRSQYNPNTQRNSGYEDTYR

NEEKNSSENNTNTDNKACALQCFLENLQMTAQDGMPDKYLVTHAITK
NVKNDDLQDFLQESIDECFQILENEDSDDKCEFSKNLLLCLSEKGKAN
CDDWKDDMHF

>MsexOBP21 MIKQMCAFLFALSVICSVNLAASKSIYVFPPEKAEMILENAIKCITESGL
QTFVGQEIKQGKYTDDERTLGALVCANEKIGYSNESGRLNIDKIMIDLF
PLKPEIRSDLEACNKDYGLDPVGTFKSFLICFRKRVPFRVVL

>MsexOBP22 MSWLHAVVALALAASAAPATTSCKNCIALGKEEKAMFRAHSDACLPQ
SGVEPKVVESMLNGQLVESAALRRHVYCVLMKCKLVSKEGKLMKNA
MLGKMAMRSDGKNATKVLEGCADQTGDTPEDLAWNLFRCGYDKKT
MLFDYMPTSGASSGDIDNISK

>MsexOBP30 MYINMDFRVKLVTRCLTLKRHSYCKMKITASLVFVLLNVVSIYGESKEI
YFFPKEAAVTFLEATLKCIADSNYDSSVIDQIMQGKYIEDDKTVNALIC
ACVATGFGYPDGKVNVEKIMKESVPTRQDLRPFIEDCNRESGKTPAQTF
RGIVKCYREKLPVQLRFSN

>MsexOBP31 MQEVEYADERRNVTDILKETDVPNSTSFNDEGFTTRNLEDAALDSTKI
QIIRKYNDTDTISRRKKRSEPLFDKPDTNQCLSQCVFANLQVVDSRGIPR
ETELWNLVQSAVTSQQSRAALRDQIRACFQELQSEAEDNGCSYSNKLE
RCLMLRFADRKMDEGNANEKKADQ

>MsexOBP32 MLCMHHCKLTIFVTTINMQSYLFLTLVLAVVGINADIVLLTDVQKEQSN
ANIAACIKESGVKPEILAEAKKGNYSEDEAMKEFLLCFFNKTGIMNAD
GKLNLDVALANLPPGVDKNEATKALEECQHKNGKDAADTAFTIFKCY
SAATKTQVLF

>MsexOBP33 MKAAQNLFGIILFSVFAAVIAASTKKIFHLSPESGEKLVEGIIKCIAKLNF
DPSLINLIKEGKYLEDERLIKAIICMNVDSGVGTADGRLNVDAVMERIFS
NNAEIRKGLICCEKEYDGTPVGNLRGTLTCLKETLPFKIRM

>MsexOBP35 MRTAIFLLLVSVISCYAAEEKDTYYYPKAPSEIFLNAAKKCVEDLSYNSS
IMDQIMQGKYIEEDKTLNVLICAAVNTGYGNADGKLNVEKVVKELYPE
RQDVWPIIEKCNLEQVTSTPLETFKGIVVCLKNNLPFKIRFPM

>MsexOBP36 MKVTILVILIIGVSCDAAFTKPIYRFPPKQSEMYVEAVVKCVAKLGYDL
AILDQIRQGKYTDDDKSVEALVCANNDIGYGLPNGQLDADKTIQDLFP
TKPEIKSVFDKCDKDYGVDPAGNFKAFLLCFKDEIPFKVII

>AipsPBP1 SQEIIKNLSLQFAKPLEDCKKEMDLSDTVITDFYNFWKEGYEFTNRQFG
CAILCLSSKLELLDQDLKLHHGKAQEFAKKHGADEAMAKQLVDMIHS
CTQSTPDVADDPCMKTLNVAKCFVAKIHDLKWAPSMDLIMGEVLAEV

>AipsPBP2 SQEVVASFSKGFTNVVEHCKAEVNAGEHIMQDIYNFWREEYQLVNRD
LGCMVLCMANKLGLIGEDQKMHHAKAEEFAKSHGADEAVAKQLVAIL
YECETKHAAVEDECGMALEIAKCFRTKMHELKWAPSMEVAMEEIMTA
V

>AipsPBP3 IEPSKDAMKYITSGFVKVLEECKQELNMNDRIIADLFHYWKLDYTLLN
RDTGCAIICMSKKLDLLDDTGRMHHGNAQEFALKHGAGEEVASKIVTII
HDCEKKFERDDDECLRVLEVAKCFRTGIHDLDWQPKVEVIVSEVFTDM

>AipsGOBP1 DVNVMKDVTLGFGQALDKCRQESDLTEEKMEEFFHFWRDDFKFEHRE
LGCAIQCMSRHFNLLTDSSRMHHVNTEEFIQSFPNGEVLARQMVALIH
GCEKQFDHEDDHCWRILHVAECFKHACVAHGVAPSMEMMMTEFIME

AEAR

>AipsGOBP2
TAEVMSHVTAHFGKALEECRDESGLSAEVLEEFQHFWREDFEVVHREL
GCAIICMSNKFSLLQDDSRMHHVNMHDYVKGFPNGEVLSGKLVELIH
NCEKQYDTLTDDCDRVVKVAACFKVDAKAAGIAPEVAMIEAVMEKY

>AipsOBP1
DVTSKCQGSKYENECDKLTCVFRKAKWLDGNAVDKAKLITYFEQFEK
DHPEWAPAMQNVKTSCLGAELKTQGVFLNCPAYDVMHCVLGSFIKHA
TPTQWSTSASCSYPRAYAAACPICPEDCFSAQVPFGSCNACYLPPRTP

>AipsOBP2
DDDANKAAFREAFKPILDECSKEHGVSNDDIDAAKKAGSADAIKPCFF
GCIYKKAEVFNAKGEYDVDSALSKLKKFVPDEAKFAKYAEIGKKCASV
NEKPVTDGDAGCERGAMLTACFLENRAEMLI

>AipsOBP3
QGLTGTDSGPPGFQRPQSYVPKHCFAPPPGVDLHTCCPIPQLFPDEDME
SCGIQKLTKEQYENPSPARIPCQESICLLRNANLLKQNNSIDYEKMGDF
VDNWAKMDPDFTIPITNAKKVCLIEGGPPAPPVCEPDRIFTCLTSYVLW
NCKLRLDSGEGCKILKEHMDGCRPFLAGP

>AipsOBP4
LTSEEEANIKEAFHPFIMKCAEEYGITEEQFEEAKEKHSAEGIDPCFMSC
FMKESGFFDSAGKFDADKTKEFVDAHLTSERAITFMEAVGSECAKVND
EEVTDGDKGCDRAKLMWGCIQDLKEKMEGSE

>AipsOBP5
NVTLPPEQSEKALKTASECIKETGVSKEVLAEAKKGHIADDEGLKKFTL
CFFKKAGIVDNDGKLNLETALAKLPPGVDKAEAKKVLEGCQAKSGKT
PQDTAFEIYKCYHAGAKTHIALAGI

>AipsOBP6
QRENKGASLKPLSVCCDIPELGDPKHLAKCSNPKLPGPCNDVQCVFEE
SGFLTDKNTLNKEAYRNHLKQWEENNKGWTVAVDKAIKECVDNDPR
QHLDIPCKAYDVFTCTGIAMLKKCPDSAWKC

>AipsOBP7
HKPNLRDAWRSELDECAKEYPVTNDEIDTAVRSGDSSNLNPCFNFCVF
NKTGFFTENGEYDLKNGLIKLRKAIRDDEEYTKFEEVATECTEDKNTSC
DEKAKCDSANRLSLCFLRFKDKVRI

>AipsOBP8
MYLRSTNGGVRSFPLGESAYTTKIVEICSKETGLKKQVPPEEKEIKFSQR
KGLREFNDCYLAKTGVTTSDGKLNIDEALEKLPPGFAKPFVEHCQANII
LGYIEENVNDFSTCFHQEVQNHLLSFYGFENYWVMLVLGTSFDKTRFT
TLFFDKHFDFWLAERAGFVNL

>AipsOBP9
VFICGVLSLNVKASSLDELKMKYVEMIIECSDTYPITAADTLQLKTKTM
PDNESIRCLFACVYKKAGMMNEQGELSVEGVNEMTRRYLSDDPDKIK
KSEQFTEACKSVNDVPVSDGTRGCDRAALIFKCTVEKSPDFDLL

>AipsOBP11
PFITKCKWDDSKCIKESAQKVIPLFADGIPDLHVEKHDPLLIKRVDASSP
NLKLIVTDIEVKGLKNCEAKKITRDLKAMKLSVKFLCAVDFKGVYDM
KGQLFVLPIEGNGDLTAHVPKIQLNAEVDMVDKTGKDGKKHWGVKS
WRHSFELKEKSNVKFENLFPDNEFLRKTTEELIASNGNDVIVEVGPEIIK
AVTAKVIESIKKLFDEVPVEELAIDE

>AipsOBP12
QMTREQVKNSGKLVKKTCSAKNDLTEDEVKDVDKGKFIEEKKFMCYV
ACVYKMGQAVKGNSLNHDMMIRQVDMLFPADMKAPVKAAIEHCRPV
AKKYKDICEASYWTAKCVYEFDPPNFMFP

>AipsOBP13
MTMKQIRNTGKMMRKSCQPKNNVEDEKIDPIAEGIFIDEPEVKCYMAC
IMKMANTLKNGKLNFDAALKQADLLLPDDIKEPAKEAIIACKKAAEGH
KDICDVSFHVTKCIYNQNPGIFYFP

>AipsOBP15	MTRQQLKNSGKILKKNCMNKHQVTEDQIGTIEKGKFVEDKKVMCYIA<br>CIYELTSVIKNNKLNYESSLRQIDIMYPADLKESAKAAVENCKDVQKKY<br>KDICEASFHTAKCMYDFKPEDFIFA
>AipsOBP16	MFPGSIPFISGCVHLGVSNYFRSTQSNLVVHYEDDQIVDAIYNCQDENG<br>FDEVLSNSTNLEENFPEKEGLKKSNDCFLKKTGFVTSDGKLNIDKTLEK<br>LPPSFVKPIVEHCQANIALNYTTESVENFSSCYHDGILNHIFAATEVGIFP<br>FIQTWKFFVPGTSFADTILILN
>AipsOBP17	MTREQVKKTMTVIKKQCMPKNSVTEDQIGKIEQGVFNEDRNVMCYVA<br>CVYKSLQVVKNERLDLGLISKQIDALYPPELKEPTKKAVSQCINIQDSY<br>NDLCEAVFHSVKCLYEKDPATFIFP
>AipsOBP18	LTDEQKEKLKKHRTECLTETKVEEALVNKLKGGDYKTESEPLKKYALC<br>MMTKSELMTKDGKFKKDVALAKVPNAADKPSVEKLIDACLANKGNT<br>PHQTAWNYVKCYHEKDPKHAIFL
>AipsOBP19	EEIKEIIQTVHDECVANTGVAEEDITNCENGIFKEDPKLKCYMFCLMEE<br>ASLVDDDGTVDYDMLVSLIPDEYYERTTKMIFACKHLDTPDKDKCQRA<br>FEVHRCSYEKDPDLYFLF
>AipsOBP20	MLVINATDYDYEGYGTGNMGEKLLTSVPRPASSSNNINNNDTSRTRRSE<br>PLLNKPDLDQCLSQCVFANLQVVDSRGIPREAELWNKVQSSVTSQQSR<br>SALHDQIRACFQELQSEAEDNGCSYSNKLERCLMLRFSDRKVEGKAST<br>PKPASTEQS
>AipsOBP21	ISEDEKKAFIEAMKPMVEECGSDCGLTEEDYKKHSKGEDMDPCFKKC<br>MMQKLGFLDEDGKYNRKQLHESISEYTGDKDEAKRVQEQLDSCFDAN<br>GDNDGDDEESQMKRVDVLFKCLKEIKE
>AipsOBP22	GVVMDEDMAELARMVRESCVDETGADVKLVEAVNGGADLMEDDKL<br>KCYIKCTMETAGMMSDGEVDIEAVMALLPPEMAEHNGPALKSCGTQR<br>GADDCDTAWKTQVCWQNANKAEYFLI
>AipsOBP23	NEDERAAFHEAAKPILVECSKENGVSFDKLKAAKEAGSADGIDPCFFSC<br>VFKKTGVFNSKGDFDLDNSLTKLKEFVSNDEDYAKVAEVGKKCESVN<br>EKDVSDGEAGCERASLLTACFLEHRAEIPV
>AipsOBP24	ATTKDAGTKEAIMTTTVANQDSSIDSNDVDVLAVMNVCNESFRIEMSYI<br>QALNESGSFVDETDKTPKCFIRCVFENVGIVSEDGRMFNPARAAVIFAG<br>ERNGKPMDDIADMTALCAADRKETCPCDRSYQFLRCLMSMEIERYEK<br>S
>AipsOBP25	SRKLREAMRPIIEQCSKEHGVTDADIQASKDSNNAASLPDCFNHCLFEK<br>SGFIDKNGRYDRDSGLKNLSKYLKDVNQYNKVVEVTKECASVEEKPA<br>TGCELGTRLTACLLDHQTSILI
>AipsOBP26	VSEEEKAVAREAMAPILAECSKAEGVSDEDIEEAKKNPSVDAVNSCFIR<br>CVMRKTDALNEKGLFDSDAALAKIRPFVKSDEDFAKFEEIGKACMSVN<br>DKEVSDGEAGCDRAKLLLACFLEHKAEMLY
>AipsOBP27	DSAISADAESRCRNPPTAPQKIERVITLCQDEIKLSILREALDVIKEEHTM<br>PAQRRRDKREVPFTHDEKRIAGCLLQCVYRKVKAVDGYGFPTLEGLVG<br>LYSDGVNERGYFMAVLEASRECLMKNHDKFSRTMPMDNGRNCDVSF<br>DIFECISDRIGEYCGTSGL
