# Supplementary material for: Pheromone-Binding Protein 1 Performs a Dual Function for Intra- and Intersexual Signaling in a Moth
Source: Int J Mol Sci. 2024 Dec 6;25(23):13125. doi: 10.3390/ijms252313125 (PMC11642448; doi:10.3390/ijms252313125)
Supplement: Supplementary file 1 [file ijms-25-13125-s001.zip › Table S3.pdf]

Table S3 Primers used for qRT-PCR and cloning of PBP genes of *Agriphila aeneociliella*

Genes	Forward primer (5’→3’)	Reverse primer (5’→3’)
**qRT-PCR**		
GAPDH	GATTGGTATCAACGGTTTCGG	CCTTCTTGGCTCCACCTTCTA
AaenPBP1	TGAGGCGACGGCAAAGAA	GAACAGCACTTCGGAGTCAGG
AaenPBP2	GCAATGCGCTTGAGTTCG	TGTCGTCTCCTGCTGGGTT
AaenPBP3	TGTCCGTGGAAGCACTTGG	AGCAGAATGAAGCGAACGAG
AaenGOBP1	GTCTGACGGAGGAGAAGATGG	GTCGGTGAGCAGGTTGAAGTAG
AaenGOBP2	ACTTCGGGAAGGCTTTGGA	TCTGGTGGACCCTGGTATCG
AaenOBP7	AAAGTTGGTGTTCGTTTTGTGG	AGAGGGGATGGTCCTTGATG
**Clone**		
AaenPBP1	AATCCGTAATACAATGCCAAGG	TGGGTCAAGATGTGGGAGAA
