# Supplementary material for: Pheromone-Binding Protein 1 Performs a Dual Function for Intra- and Intersexual Signaling in a Moth
Source: Int J Mol Sci. 2024 Dec 6;25(23):13125. doi: 10.3390/ijms252313125 (PMC11642448; doi:10.3390/ijms252313125)
Supplement: Supplementary file 1 [file ijms-25-13125-s001.zip › Table S4.pdf]

Table S4 Compounds for fluorescence binding assay and molecular docking

Ligand	CAS NO.	purity (≥%)	Total score of molecular docking
(Z)-9-Hexadecenyl acetate	34010-20-3	90	10.54
(Z,Z,Z)-9,12,15-Octadecatrienal	2423-13-4	90	6.38
1-Nonanal	124-19-6	96	10.26
Heptanal	111-71-7	98	4.71
Hexaldehyde	66-25-1	99	4.25
Linalool	78-70-6	98	5.35
β-Caryophyllene	87-44-5	90	5.39
Phenethyl alcohol	60-12-8	99	3.82
3-Hexanol	623-37-0	98	5.09
1-NPN	90-30-2	98	
