# Supplementary material for: Pheromone-Binding Protein 1 Performs a Dual Function for Intra- and Intersexual Signaling in a Moth
Source: Int J Mol Sci. 2024 Dec 6;25(23):13125. doi: 10.3390/ijms252313125 (PMC11642448; doi:10.3390/ijms252313125)
Supplement: Supplementary file 1 [file ijms-25-13125-s001.zip › Table S5.pdf]

Table S5 PBPs and pheromones of lepidopteran species

Subfamily	Family	Species	PBP	Female pheromone(s)
Bombycoidea	Bombucidae	*Bombyx mori*	BmorPBP	E10,Z12-16:OH<br>E10,Z12-16:Ald<br>E10,Z12-16:OAc
Bombycoidea	Lasiocampidae	*Dendrolimus tabulaeformis*	DtabPBP	Z5,E7-12:OH<br>Z5,E7-12:OAc
Bombycoidea	Saturniidae	*Antheraea polyphemus*	ApolPBP	E6,Z11-16:OAc<br>E6,Z11-16:Ald
Bombycoidea	Sphingidae	*Manduca sexta*	MsexPBP	E10,Z12-16:Ald<br>E10,E12,Z14-16:Ald
Cossoidea	Cossidae	*Eogystia hippophaecolus*	EhipPBP	
Gelechioidea	Gelechiidae	*Pectinophora gossypiella*	PgosPBP	Z7,Z11-16:OAc<br>Z7,E11-16:OAc
Gelechioidea	Gelechiidae	*Sitotroga cerealella*	ScerPBP	Z7,E11-16:OAc
Gelechioidea	Stathmopodidae	*Atrijuglans hetauhei*	AhetPBP	Z8-13Ac
Geometroidea	Geometridae	*Ectropis obliqua Prout*	EoblPBP	Z3,epo6,Z9-18:H<br>Z3,Z6,Z9-18:H<br>Z3,epo6,Z9-19:H
Geometroidea	Geometridae	*Ascotis selenaria*	AselPBP	epo3,Z6,Z9-19:H<br>Z3,Z6,Z9-19:H
Geometroidea	Geometridae	*Semiothisa cinerearia*	ScinPBP	epo3,Z6,Z9-17:H<br>Z3,Z6,Z9-17:H
Noctuoidea	Noctuidae	*Spodoptera litura Fabricius*	SlitPBP	Z9,E11-14:OAc<br>Z9,E12-14:OAc
Noctuoidea	Noctuidae	*Heliothis virescens F.*	HvirPBP	Z11-16:Ald<br>Z9-14:Ald
Noctuoidea	Noctuidae	*Helicoverpa armigera Hübner*	HarmPBP	Z11-16:Ald<br>Z11-14:Ald<br>Z9-16:Ald
Noctuoidea	Noctuidae	*Spodoptera exigua Hübner*	SexiPBP	Z9,E12-14:OAc<br>Z9,E12-14:OH<br>Z9-14:OH<br>Z11-16:OAc
Noctuoidea	Noctuidae	*Helicoverpa assulta Guenée*	HassPBP	Z9-16:Ald<br>Z9-16:OAc<br>Z11-16:Ald<br>Z11-16:OAc
Papilionoidea	Papilionidae	*Papilio machaon*	PmacPBP	
Papilionoidea	Pieridae	*Pieris rapae*	PrapPBP	methyl palmitate

Pyraloidea	Crambidae	*Agriphila aeneociliella*	AaenPBP	Z9-16:OAc<br>Z9,Z12,Z15-16:Ald
Pyraloidea	Crambidae	*Ostrinia nubilalis*	OnubPBP	Z11-14:OAc<br>E11-14:OAc
Pyraloidea	Crambidae	*Ostrinia furnacalis*	OfurPBP	Z12-14:OAc<br>E12-14:OAc
Pyraloidea	Crambidae	*Diaphania indica*	DindPBP	E11-16:Ald<br>E10,E12-16:Ald
Pyraloidea	Crambidae	*Loxostege sticticalis*	LstiPBP	
Pyraloidea	Crambidae	*Cnaphalocrocis medinalis*	CmedPBP	Z11-16:OAc<br>Z13-18:OAc<br>Z13-18:Ald<br>Z13-18:OH<br>Z11-18:Ald<br>Z11-18:OH
Pyraloidea	Crambidae	*Chilo suppressalis*	CsupPBP	Z11-16:Ald<br>Z13-18:Ald<br>Z9-16:Ald
Pyraloidea	Pyralidae	*Orthaga achatina*	OachPBP	Z11-16:OAc<br>Z3,Z6,Z9,Z12,Z15-23:H
Pyraloidea	Pyralidae	*Dioryctria abietella*	DabiPBP	Z9,E11-14:OAc<br>Z3,Z6,Z9,Z12,Z15-25:H
Tineoidea	Psychidae	*Eumeta japonica*	EjapPBP	
Tortricoidea	Tortricidae	*Choristoneura rosaceana*	CrosPBP	Z11-14:OAc<br>E11-14:OAc<br>Z11-14:OH<br>Z11-14:Ald
Tortricoidea	Tortricidae	*Argyrotaenia velutinana*	AvelPBP	Z11-14:OAc<br>E11-14:OAc<br>12:OAc
Tortricoidea	Tortricidae	*Epiphyas postvittana*	EposPBP	E11-14:OAc<br>E9,E11-14:OAc<br>E11-14:OH<br>E11-16:OAc
Yponomeutoidea	Argyresthiidae	*Argyresthia conjugella*	AconPBP	
Yponomeutoidea	Plutellidae	*Plutella xylostella*	PxylPBP	Z11-16:OAc<br>Z11-16:Ald
Yponomeutoidea	Yponomeutidae	*Yponomeuta*	YcagPBP	Z11-16Ac

		cagnagella	
Zygaenoidea	Zygaenidae	*Phauda flammans*	PflaPBP

>AaenPBP1
MWEKMGIKMFVVVLLGMSVSVDSSQTVVKSMTKYFFKAYEVCTKEYNIKEGTLGQIFNFWREDF
TTNSRDIGCTIYCLSTKLDLLDPEGKLHHGNAAEFAMQHGSDEATAKKLVEILHTCEQTTPPNDDK
CMKALDVAFCFKKELHRLDWAPDSEVLFEEIIAELG
>BmorPBP
MSIQGQIALALMVYMAVGSVDASQEVMKNLSLNFGKALDECKKEMTLTDAINEDFYNFWKEGYE
IKNRETGCAIMCLSTKLNMLDPEGNLHHGNAMEFAKKHGADETMAQQLIDIVHGCEKSTPANDD
KCIWTLGVATCFKAEIHKLNWAPSMDVAVGEILAEV
>SlitPBP
MANARWRFVFVVYALYLTSAVLGSQDLMAKMTKGFTRVVDDCKTELNVGDHIMQDMYNYWRE
DYQLINRDMGCMLLCMAKKLDLMDDQTMHHGKTEDFAKSHGADDDVAKKLVSVIHECEQQHT
GIADDCMRVLEVAKCFRTKIHELKWAPSMEVIMEEVMTAV
>HvirPBP
MMSVRLMLVVAVWLCLRVDASQDVMKNLSMNFAKPLEDCKKEMDLPDSVTTDFYNFWKEGYEF
TNRHTGCAILCLSSKLELLDQEMKLHHGKAQEFAKKHGADDAMAKQLVDMIHGCSQSTPDATDD
PCMKALNVAKCFKAKIHELNWAPSMELVVGEVLAEV
>HarmPBP
MEFHRSTMMSVRLALVVAVCLFIRVDASQDVIKNLSMNFAKPLEDCKKEMDLPDSVTTDFYNFWK
EGYEFTNRQTGCAILCLSSKLELLDQELKLHHGKAQEFAKKHGADDAMAKQLVDLIHGCAQSTPD
VADDPCMKTLNVAKCFKAKIHELNWAPSMELVVGEVLAEV
>SexiPBP
SQELMMKMTKGFTKVVDDCKAELNAGEHIMQDMYNYWREDYQLINRDLGCMILCMAKKLDLM
EDQKMHHGKTEEFAKSHGADDEVAKKLVSIIHECEQQHAGIADDCMRVLEISKCFRTKIHELKWAP
NMEVIMEEVMTAV
>EoblPBP
RECSQEVMHKITKDFALVLEDCKKQENVGDHIMQDIFNFWHEEYALVNPELGCVMLCMAGKLDL
MDGDDMHHGNAHEFAKKHGADDDLAKQLVTMIHDCEKASASIADRCARALETTKCFRGKIHGL
KWAPSMRVIMEEVMADMNV
>HassPBP
MNFAKPLEDCKKEMDLPDSVTTDFYNFWKEGYEFTNRQTGCAILCLSSKLELLDQEMKLHHGKA
QEFAKKHGADDAMAKQLVDLIHGCSRSTPDVTDDPCMKALNVAKCFKAKIHELNWAPSMDLVVG
EVLAEV
>OachPBP
MSLLVRVVALILAYSAIYFGVDSSADIIRDMTVNFGKALDTCKTELDLPESINADFNNFWKEGYELN
NRLTGCAIMCLASKLDLLDPTGSLHHGNANEFAKKHGADEAMAKQLVDLIHGCEKSAEPDKDAC
VTALNIAKCFKVEIHKLNWAPSMDLIVGEMLAEV
>PgosPBP
MAEMWKISMLVLVYMAIDSRVETSQDVMKTMSMNFAKALDACKKEMELPDSIDVDFNNFWKED
YEIXNRFTGCAIMCLSTKLDLVSPDGSLHHGNAQEFAKKHGADDAMAKQLVDLLHGCEKSAPDN
EDGCMKVLGIAKCFKTEIHKLNWAPXVEVVVGEILSEV
>CrosPBP

MLQQKELLLFAVVCLSLTQMVEPSADVVKGMTLNFGKGLEECKKEMNLPDSINADFYNFWKDDH
VLTNRDTGCAIMCLSSKLELVSDGKLHHGNTLEYAKQHGADDTVAQQLVDLIHNCEKALPDLEDP
CMKVLEWAKCFKIEIHKLNWAPSMDVLAGEMLAEI
>AvelPBP
MLKQNKLVLFAVVYLAIQQKVESSQDVIKGMTLNFRKGLDECKKEMNLPDSINADFYNFWKDDH
VLSNRDTGCAIMCLSSKLELVSDGKLHHGNTFDYAKQHGADETVAQQLVDLIHSCEKSLPDLEDPC
MKVLEWAKCFKTEIHKLNWAPSVEVLAAEMLAEV
>YcagPBP
MALLTRWRMLALIAACLAVRLDWAMASQDIMKKLTVGFSKALDQCKTELAIQENVLQDFYNFWR
EDYTLVNREMGCVLMCMASKFDLITEDMKVHHKNAHEFAKTHGADDEMAKQLVSMIHECEKTH
EGVVDDCGRVLEMAKCFKTKIHELKWAPSMEVVLEEVMTEIQS
>OnubPBP
MGLSLRLLVVVAAAILGAECSQDVMKQMTINFGKALDTCRKELDLPDSINADFYNFWKEGYELSN
RQTGCAIMCLSSKLDLVDPEGKLHHGNTHEFAKKHGADDSMAKQLVELIHKCEGSVADDPDACM
KVLNIAKCFKAEIHKLNWAPSMDLIVAEVLAEV
>OfurPBP
MGLSLRLLVVVAAAIFGAESSQDVMKQMTXXFGKALDTCRKELDLPDSINADFYNFWKEGYELS
NRHTGCAIMCLSSKLDLVDPEGKLHHGNTHEFAKKHGADDSMAKQLVELIHKCEGSVADDPDAC
MKVLNIAKCFKAEIHKLNWAPSMDLIVAEVLAEV
>EposPBP
MMNHKELVLFAVVCLSLYQAVEPSQDVVKDMSLNFRKGLDACKKELNLPDTINADFNRFWNDDH
VVTNRDTGCAIMCLSSKLELVTDTGLHHGNTLEYAKQHGADETVAQQIVDLLHTCAQAVPDLQDT
CMKVLEWAKCFKAEIHKLNWAPSAEVMAAEMLAEV
>ApolPBP
MLRKISLLLLPVFVAINLVHSSPEIMKNLSNNFGKAMDQCKDELSLPDSVVADLYNFWKDDYVMT
DRLAGCAINCLATKLDVVDPDGNLHHGNAKDFAMKHGADETMAQQLVDIIHGCEKSAPPNDDKC
MKTIDVAMCFKKEIHKLNWVPNMDLVIGEVLAEV
>AselPBP
MQRSWCFYCRLLVVLGVFLALVQERECSQEVMHNLSKGFAEVLEDCKKQENVGDHIMQDFYNF
WHEEYSLVNREMGCIILCMAGKLDLLDGDTMHHGNAHEFAKKHGADDALAKQLVGLVHECEQA
SASVEERCARALETTKCFRGKIHGLKWAPSMRVVMEEVMADM
>MsexPBP
MKVAVVAIVVYLAVGNVDSSPEVMKNLCLNFGKALDECKAEMNLSDSIKDDFANFWVEGYEVSN
RDTGCAILCLSKKLDMIDPDGKLHHGNAMEFAKKHGADEAMAKQLLDIIHNCENSTPPNDDACL
KTLDIAKCFKKEIHKLNWAPNMDLVVGEVLAEV
>PxylPBP
MVKMITKKPACLMMVLMCALKKVESSADVMKGLSENFGKALGDCKKELDLPDSIMTEFYNFWK
DDYVLSDRSTGCAIICLSSKLDLLDPDGNLHHGNAKDFALKHGADEGMAGQLVGMIHECEKAAP
DNPDACLKVLDIANCFKKKIHELKWAPSMDVVVAEVLADV
>DindPBP
MWVKMILITVAVVMMSVKVDLSQTLLKDMTKNFLKAYEQCQQELSLPESTAKELMFFWKDGYEV
SSREAGCTILCLSKKMDLIDPEGKLHKGKSADFVKQHGTDDETANKVVDILHTCESNAAPNDDHC
MVALAVALCFKKEIHNLNWAPDPEVILEELMAEMS

>ScinPBP
MEWKHVLVVVVLVTVRRAEGGDAMKLLATGFVKVLEECKKELNLDDNLISDLYHYWKLDFSLL
QRETGCAIICMSKKLELLGEDGKLHHGNAKEYAMRNGAGDELATKMISIIHGCEDKGEGIEDDCA
RVLEVAKCFRVGLHDLHWEPKVDVIITEVLTEI
>EhipPBP
MLTQTKIVVLVIVYLAIDSRVESSKEIMKEITVNFGKALADCKREMELPDSIDVDFYNFWKEDYEVS
NRYTGCAIICLSTKLDLVDPDGGLHHGNAHEFAKKHGADDGMAKQLIDIIHQCEKSTPRNDDGCIM
MLGIAKCFKAEIHKLDWAPSMDLMVGEVLAEV
>ScerPBP
MAEMWKITVVVLVYLAIDSRVESSQEVIKTMSIGFAQALDSCKKEMELPDSIDVDFANFWKEGYEI
TNRFTGCAIICLSTKLDLVSPDGSLHHGNAQDFAKKHGADDAMAKQLIDMIHGCEKSAPAIEDGC
MKILAIAKCFKMEIHKLNWAPTAEVVMGEILAEA
>EjapPBP
MDTHNSRVPSTWGRKRISDEGSGPLKLAFIGQNVTAKAATSRVKSLDLHEDAYNHLKNYWDEKFE
LVNRDFGCLIICMSKKLDLIDEDGKLHHGKAKEFATAHGAVGLAHELDKYNVLNERDSGIEVRSRL
RSHARPADEETAQQLIDIVHNCEKQTSGGDDPCATMVEVAKCFRIKIHELKWVPSMEILLDEGIKIR
RNEESSSETENRLSIHRQYVAQSRARESSTERSQRLAEQNMRTAQIRARESSLQRSQRLAEQNESSS
ERFQRLQDQRERQQTSRARSRNQVLAHSNRSAFRYDPQIDYAQQSSVQIGDMNKICPKCSAKKWV
DETNGMCCASGKRLRAEEYIHLRDALNQDGNVDPSNIGQRVILPYSFTGSPRYLHEKTQDAMTYV
RNYGRPDLFVTFTCNPEWPEIKAELLPDQRSFDRHDIISRVFHLKMKGMLEIVSLMDY
>LstiPBP
MGFSVRLLVVLVAVTIYGVNSSQDIIKQMTINFGKALDSCRKELDLPDSINADFYNFWKEGYELSNR
QTGCAIMCLSSKLDLVDPEGKLHHGNTHEFAKKHGADDAMAKQLVDLIHKCESDVPDDPDPCLK
VLNIAKCFKAEIHKLNWAPSMDLIMAEVLAEV
>PmacPBP
MEKKIIFVVIVCLTTCKTVYSSQEIIQTMSINYMKGLDTCKSELNLPDVVDIEFAQFWREDYIISNRL
TGCAIVCLSSKLDLLEPDGSLHHGNAADFAKKHGADEAMAQQLIDILHQCEQQYPDKMDACLYAL
QVCNCFKTQIHKLNWAPDVELIVGEVLAEI
>DabiPBP
MSLSVLVLAVVAACLAGVDSSSADIMKDLTANFGKALSECKKELDLPDSINADFYNFWKEDYELS
NRFTGCAIMCLSTKLELVDSEGKLHHGNAHDFAKKHGADDDMAKQLVDLIHGCEKSVPPNEDAC
LNVLEIAKCFKKEIHKLNWAPDMDLIVGEVLG
>AhetPBP
MAASSKWRVMMVAVLLSTMTQVLGSQEIMKKLTLGFSKALDQCKKEMNIQDHIMQDFYNFWRE
EYSLVNREMGCAMLCLASKLDLVTDDNKLHHTNAREFAKSHGADDDLAKQLIDMIHECEKVHEG
IQDDCVKVLEIAKCFRTKIHDLKWAPSMETVLEEVMTEIQ
>DtabPBP
MTKTYTFLAVAIVLLAIDSRVDSSQDVMKDLSVKFGESMNQCIKEMDLPDVSADFYNYWKEDFVI
TRRETGCLFSCLAKKVSMQHSDGLLHKDNTHNFATKHGADDEMAAKLVETIHACENSISESDDCV
RVLSIANCFKKEMHKLNWAPSAELVTQELMTIL
>CmedPBP
MWAKTLMVVVTVVMMSVNVESSQTLLKDMTKNFLKAYGQCQKELGLPDSTATELMNFWKEGY
EIKSREAGCAIMCLSKKLEVIDPEGKLHKGKTTEFIVAHGTDEATAHKLIDILHACMQSVTPSEDHC

LMSLQVAMCFKAEIHKLGWAPDTELLFEEMVAEMQ
>CsupPBP
MVRDTMMLKLVVVMCLTMTVVVDSSQTVMKSMTKNFLKAYEVCAKEYSLKEGTAGILIGFWKD
DFSTTSRDVGCAILCLSTKLDLIDPEGKLHHGKATEFAMQHGSGEEMAKKLVEILHNCEQTVTPNE
DKCMRALDIAMCFKKELHTLGWAPDPELLFEELIAEMR
>PrapPBP
MACYAYILCVLTLFSSALASQETLKNIAHSFLKVLDECKQELNLHENILLDLYHFWKEDYGLLKRD
TGCAIMCMSQKLQLVDTSGNLHHGNAQEFAVAHGADEEVAQKLVNMVHECEKQHQVKEDLCER
ALEVAKCFRSGIHLLKWTPTVEVLVGEVLTEV
>PflaPBP
MRKFLILVGIVVLHVDNNRVQGSQDIMKDLTIQFGKALSTCKKELDLPDTIMADFKNFWNDGYEL
SNRFTGCAIMCLSSKLDLLDPEGKLHHGNAQEFAMKHGADATMAKQLTDLIHNCEKSITPTEDDCI
NVLEVAKCFKAEIHKLNWAPNMDLIVGELLAEA
>AconPBP
MAGFRPNWRLIAVFALILVLKLDRTSASQEVMKKLTAQFSTALDACKKELNIQDHILQDFYNFWRE
DYALVNREMGCVIMCMAVQLDLITEDLKMHHGKAHEFAKTHGADDELAKQLVSMIHECEKLHSG
QGTDECATTLEIAKCFRSKIHDLKWAPSMEVVLEEVMTEM
