# Supplementary figures and images for: Pheromone-Binding Protein 1 Performs a Dual Function for Intra- and Intersexual Signaling in a Moth
Source: Int J Mol Sci. 2024 Dec 6;25(23):13125. doi: 10.3390/ijms252313125 (PMC11642448; doi:10.3390/ijms252313125)

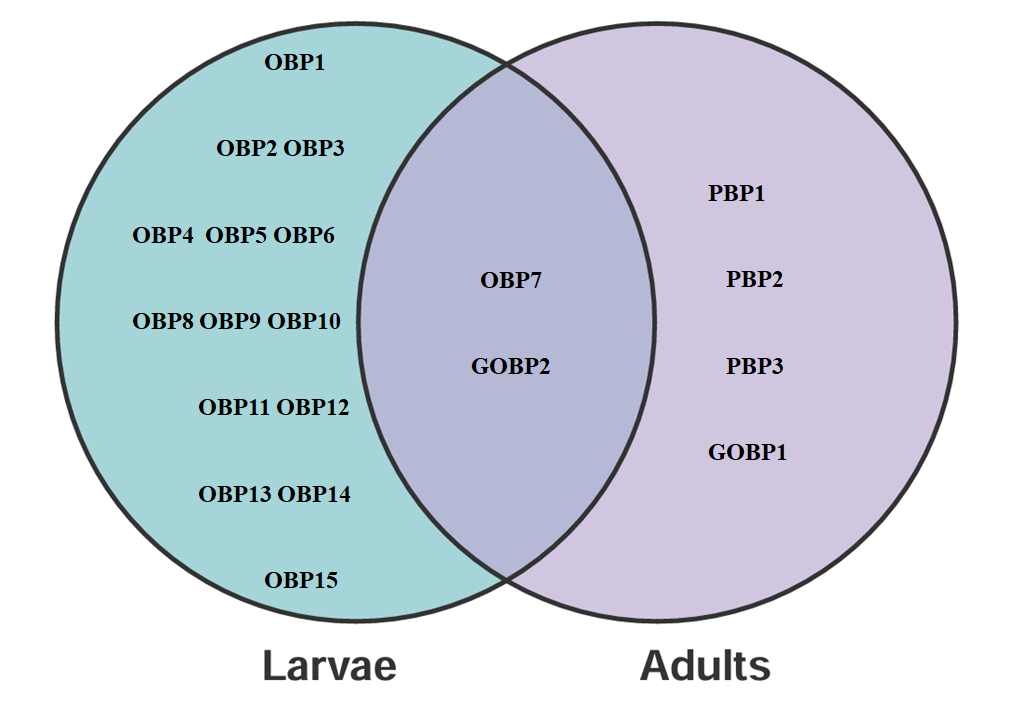

Supplement: Supplementary file 1 [file ijms-25-13125-s001.zip › Figure S1.tif]

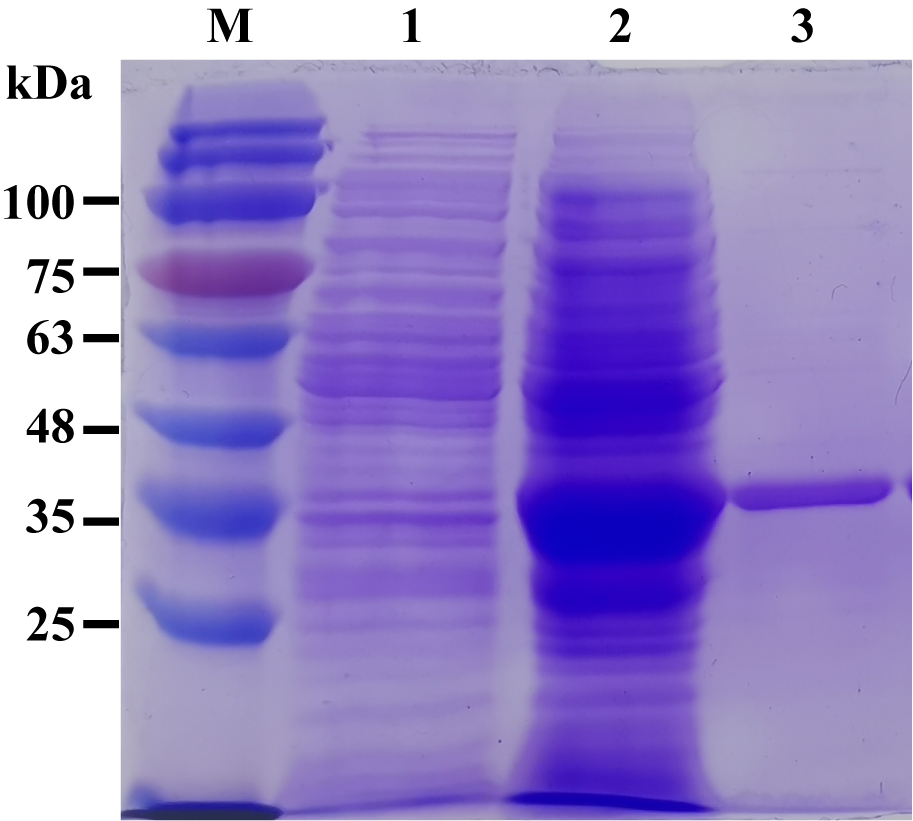

Supplement: Supplementary file 1 [file ijms-25-13125-s001.zip › Figure S2.tif]
